# Cybermycelium: a reference architecture for domain-driven distributed big data systems

**DOI:** 10.3389/fdata.2024.1448481

**Published:** 2024-11-05

**Authors:** Pouya Ataei

**Affiliations:** School of Engineering, Computer and Mathematical Sciences, Auckland University of Technology, Auckland, New Zealand

**Keywords:** big data reference architecture, big data architecture, big data systems, big data software engineering, distributed systems, decentralized system, reference architecture, domain-driven design

## Abstract

**Introduction:**

The ubiquity of digital devices, the infrastructure of today, and the ever-increasing proliferation of digital products have dawned a new era, the era of big data (BD). This era began when the volume, variety, and velocity of data overwhelmed traditional systems that used to analyze and store that data. This precipitated a new class of software systems, namely, BD systems. Whereas BD systems provide a competitive advantage to businesses, many have failed to harness the power of them. It has been estimated that only 20% of companies have successfully implemented a BD project.

**Methods:**

This study aims to facilitate BD system development by introducing Cybermycelium, a domain-driven decentralized BD reference architecture (RA). The artifact was developed following the guidelines of empirically grounded RAs and evaluated through implementation in a real-world scenario using the Architecture Tradeoff Analysis Method (ATAM).

**Results:**

The evaluation revealed that Cybermycelium successfully addressed key architectural qualities: performance (achieving <1,000 ms response times), availability (through event brokers and circuit breaking), and modifiability (enabling rapid service deployment and configuration). The prototype demonstrated effective handling of data processing, scalability challenges, and domain-specific requirements in a large-scale international company setting.

**Discussion:**

The results highlight important architectural trade-offs between event backbone implementation and service mesh design. While the domain-driven distributed approach improved scalability and maintainability compared to traditional monolithic architectures, it requires significant technical expertise for implementation. This contribution advances the field by providing a validated reference architecture that addresses the challenges of adopting BD in modern enterprises.

## 1 Introduction

The rapid advancement of digital technologies and the ubiquity of internet-connected devices have ushered in an era of unprecedented data generation and connectivity. This digital age is characterized by the explosive growth of data, often referred to as “Big Data,” which has transformed the landscape of data processing and management. BD, with its immense volume, variety, and velocity, holds the potential to revolutionize decision-making processes, enhance operational efficiency, and drive innovation across various domains (Ataei and Litchfield, 2020; Rad and Ataei, 2017).

The value of BD lies in its ability to uncover hidden patterns, correlations, and insights that can lead to data-driven strategies and competitive advantages. Organizations that effectively harness the power of BD can gain a deeper understanding of customer behavior, optimize supply chain processes, improve risk management, and identify new business opportunities (Popovič et al., 2018; Chen et al., 2012; Ataei et al., 2024). However, despite the immense potential of BD, many organizations struggle to successfully integrate it into their existing structures and realize its full benefits.

One of the critical challenges in BD adoption is the development of robust and scalable data architectures. Current RAs often fall short in addressing the dynamic and complex nature of BD, grappling with issues of scalability and efficiency as data ecosystems continue to expand (Gorton and Klein, 2015; Nadal et al., 2017). The monolithic nature of these architectures and their lack of comprehensive support for cross-cutting concerns hinder their ability to adapt to the ever-evolving BD landscape (Ataei and Litchfield, 2023).

Recent surveys highlight the prevalence of these challenges in BD implementation. A report by Databricks reveals that a mere 13% of organizations excel in their data strategy (Technology Review Insights in Partnership With Databricks, 2021), while NewVantage Partners finds that only 24% have successfully become data-driven, with a mere 30% possessing a well-established BD strategy (Partners, 2021). These findings are further corroborated by reports from McKinsey and Company (Analytics, 2016) and Gartner (Nash, 2015), underscoring the difficulties organizations face in successfully integrating BD into their operations. Among the challenges, data architecture, organizational culture, and lack of talent are highlighted.

To address these challenges of data architecture and bridge the gap between the potential of BD and its successful implementation, this study introduces Cybermycelium, a domain-driven, distributed RA for BD systems. Cybermycelium aims to overcome the limitations of current RAs by incorporating domain-driven design principles and distributed computing concepts from contemporary software engineering (Ataei and Staegemann, 2023). By emphasizing scalability, maintainability, and adaptability, Cybermycelium seeks to provide a flexible and resilient framework for BD systems, enabling organizations to harness the full potential of their data assets.

The proposed RA adopts a modular and decentralized approach, allowing for the logical separation of data into domains with clearly defined boundaries and event-driven communication. This domain-driven architecture promotes loose coupling and high cohesion, facilitating the development of scalable and maintainable BD systems. By leveraging distributed computing principles, Cybermycelium enables the efficient processing and analysis of large-scale datasets across multiple nodes, ensuring optimal resource utilization and performance.

## 2 Background

In this section, a brief discussion of what is known about BD architectures is provided, articulating the research gap, problems that need addressing, and the objective of this research.

### 2.1 Big data architectures: state of the art

The available body of knowledge and the knowledge from practice highlight three generations of BD architectures;

1. Enterprise data warehouse: this is perhaps one of the oldest approaches to business intelligence and data crunching and existed even before the term “Big Data” was coined (Leonard, 2011). Usually developed as proprietary software, this data architecture pivots on the enterprise data warehouse, extract, transform, and load (ETL) jobs, and data visualization software such as Microsoft Power Business Intelligence (BI). As the data sources and consumers grow, this architecture suffers from hard-to-maintain ETL jobs and visualizations that can be created and understood by a certain group of stakeholders, hindering the positive impact of data on business. This also means that new transformations will take longer to be added to the workload, the system is monolithic and hard to scale, and only a few groups of hyper-specialized individuals are able to operate the system (Ataei and Litchfield, 2022).
2. Data lake: to address the challenges that occurred in the first generation of data architectures, a new BD ecosystem emerged. This new ecosystem revolved around a data lake, in a way that there are not as many transformations on the data initially, but rather everything is dumped into the data lake and retrieved when necessary. Although data lake architecture reached some level of success in comparison with the first generation of data architectures, it still falls short of being optimal. As data consumers and data providers grow, data engineers will be challenged to avoid creating a data swamp (Brackenbury et al., 2018), and because there is usually no concept of data owner, the whole stack is usually operated by a group of hyper-specialized data engineers, creating silos and barriers for gradual adoption. This also means various teams' concerns will often go into data engineer backlogs through an intermediary such as a business analyst, and they will not be in control of how and when they can consume the data they desire. Furthermore, data engineers are usually oblivious to the semantics and value of the data they are processing; they simply do not know how useful that data are or which domain it belongs to. This will over time decrease the quality of data processing, result in haphazard data management, and make maintenance and data engineering a complicated task (Ataei and Litchfield, 2023).
3. Cloud based solutions: given the cost and complexity of running a data lake on-premise alongside the whole data engineering pipeline and the substantial talent gap currently faced in the market (Rada et al., 2017), the third generation of BD architectures tends to revolve around as-a-service or on-demand cloud-based solutions (Rad and Ataei, 2017). This generation of architecture tends to lean toward stream processing with architectures such as Kappa or Lambda (Lin, 2017), or frameworks that unify batch and stream processing such as Apache Beam (Apache Beam, 2022) or Databricks (Databricks, 2022). This is usually accompanied by cloud storage such as Amazon S3 and streaming technologies such as Amazon Kinesis. Although this generation tends to solve various issues regarding the complexity and cost of data handling and digestion, it still suffers from the same fundamental architectural challenges. It does not have clear data domains; a group of siloed, hyper-specialized data engineers are running them, and data storage through a monolithic data pipeline soon becomes a chokepoint (Ataei and Litchfield, 2020; Ataei and Staegemann, 2023).

To discuss the integral facets that embroil these architectures, one must look at the characteristics of these architectures and the ways in which they achieve their ends. Most of these architectures and RAs use a monolithic data pipeline design with four key components: data consumers, data processing, data infrastructure, and data providers.

The process of turning data into actionable insights in these architectures usually follows a similar lifecycle: (1) Data ingestion: system beings to ingest data from all corners of the enterprise, including transactional, operational, and external data; (2) Data transformation: data captured from the previous step is then cleansed for duplication and quality and potentially scrubbed for privacy policies. These data then go through a multifaceted enrichment process to facilitate data analysis, (3) Data serving: at this stage, data are ready to be served to a diverse array of needs, ranging from machine learning to marketing analytics, business intelligence to product analysis, and customer journey optimisation.

The lifecycle depicted is indeed a high-level abstract view of prevalent BD systems. However, it highlights an important matter: these systems are all operating underlying monolithic data pipeline architecture that tends to account for all sorts of data in one architectural construct. This means that data that logically belong to different domains are now all lumped together and crunched in one place, making maintainability and scalability a daunting task (Dehghani, 2019).

While architectures in software engineering have gone through a series of evolutions in the industry, adopting more decentralized and distributed approaches such as microservices architecture, event-driven architectures, reactive systems, and domain-driven design (Alshuqayran et al., 2016), data engineering, and in specific, BD ecosystems, do not seem to be adopting many of these patterns. Evidence collected from the studies of Ataei and Litchfield (2022) has proven that attention to decentralized BD systems, metadata, and privacy is deficient. Therefore, the whole idea of “monolithic data pipeline architecture with no clearly defined domains and ownership” brings significant challenges to the design, implementation, maintenance, and scaling of BD systems.

### 2.2 Why reference architecture?

To justify why RA has been chosen as the suitable artifact, first it is necessary to clarify two assumptions: (1) Having a sound software architecture is essential to the successful development and maintenance of software systems (Len Bass, 2021). (2) There exists a sufficient body of knowledge in the field of software architecture to support the development of an effective RA (Ataei and Litchfield, 2020).

One of the focal tenets of software architecture is that every system is developed to satisfy a business objective and that the architecture of the system is a bridge between abstract business goals and concrete final solutions (Len Bass, 2021). While the journey of BD can be quite challenging, the good news is that a software RA can be designed, analyzed, and documented, incorporating best practices, known techniques, and patterns that will support the achievement of business goals. In this way, the complexity can be absorbed and made tractable.

Practitioners of complex systems, software engineers, and system designers have been frequently using RAs to have a collective understanding of system components, functionalities, data flows, and patterns that shape the overall qualities of the system and help further adjust it to the business objectives (Cloutier et al., 2010; Kohler and Specht, 2019).

A RA is an amalgamation of architectural patterns, standards, and software engineering techniques that bridge the problem domain to a class of solutions. This artifact can be partially or completely instantiated and prototyped in a particular business context together with other supporting artifacts to enable its use. RAs are often created from previous RAs (Ataei and Litchfield, 2020). Based on the premises discussed and taking all of them into consideration, RAs can facilitate the issues of BD architecture and data engineering because they promote adherence to best practices, they can capture cross-cutting concerns, they can serve as organizational memory around design decisions, and they can act as a blueprint in the portfolio of data engineers and data architects.

## 3 Related work

The application of RAs to address challenges in data architecture is well-established, with notable contributions from both governmental agencies (e.g., NIST's BD RA (NBDRA) (Chang and Boyd, 2018)] and industry leaders [e.g., IBM (Quintero and Lee, 2019) and Microsoft (Levin, 2013)]. Conceptual RAs have also been proposed (Maier et al., 2013; Suthakar, 2017; Chang and Mishra, 2015), and numerous domain-specific RAs have been developed, spanning fields such as national security (Klein et al., 2016) and the Internet of Things (IoT) (Weyrich and Ebert, 2015).

However, while some RAs, such as the NBDRA, are comprehensive, most are published as brief papers or white papers lacking in detail. In addition, while efforts such as Neomycelia (Ataei and Litchfield, 2021) and Phi (Maamouri et al., 2021) explore microservice architecture for BD systems, the majority of existing RAs remain monolithic and centralized (Ataei and Litchfield, 2022).

This research extends current work by addressing these limitations through a novel domain-driven distributed architecture for BD systems. This approach emphasizes the logical separation of data into domains via event-driven communication with clearly defined boundaries (Ataei and Litchfield, 2021).

## 4 Research methodology

The research methodology of this study is made up of two major phases. First, the body of knowledge in academia and industry is explored to identify architecturally significant requirements (ASR) for BD systems, and second, the chosen methodology for developing the artifact is delineated.

### 4.1 Requirement specification

Architecture aims to produce systems that address specific requirements, and one cannot succeed in designing a successful architecture if requirements are unknown (Len Bass, 2021). Therefore, in this section, the software and system requirements necessary for the development of Cybermycelium are defined. The aim is to present three integral pieces of information: (1) type of requirements; (2) approach for categorization of requirements; and (3) method for presentation of the requirements.

#### 4.1.1 Type of requirements

System and software requirements vary in complexity, from simple sketches to formal specifications. Existing classifications of software requirements were reviewed to establish the most suitable type for this study.

In reviewing classifications of software requirements, we considered multiple approaches. Sommerville (Sommerville, 2011) categorizes requirements into user requirements, system requirements, and design specifications. While this classification is widely recognized, we ultimately chose to follow Laplante's framework (Laplante, 2017), which divides requirements into functional, non-functional, and domain categories. Laplante's approach was deemed more suitable for our study due to its alignment with our research objectives and its clear distinction between functional and non-functional aspects of the system.

The requirements for Cybermycelium focussed on both functional aspects of BD processing and non-functional qualities such as scalability, modifiability, and performance. While domain-specific requirements were considered, the study aimed to develop a general-purpose BD architecture applicable across various sectors.

#### 4.1.2 Categorizing requirements

The categorization process for Cybermycelium requirements incorporated a methodical approach, primarily employing the well-recognized 5Vs model of velocity, veracity, volume, variety, and value (Bughin, 2016; Rad and Ataei, 2017). This model, pertinent to BD characteristics, was adapted to align with the specific needs and context of this study. In addition, Security and Privacy (SaP) were added as they are important cross-cutting concerns. The methodology employed facilitated a focussed categorization of requirements, which was central to the development of the RA.

#### 4.1.3 Present requirements

Upon determining the type and category of requirements, a rigorous approach to presenting these requirements was sought. Various methods are used in software and system requirement representation, including informal, semiformal, and formal methods. For the purposes of this study, the informal method was chosen. This method is well-established in both industry and academia (Kassab et al., 2014). Moreover, this approach adheres to the guidelines outlined in the ISO/IEC/IEEE standard 29148 (ISO/IEC/IEEE 29148:2018, 2018) for representing functional requirements and draws inspiration from the Software Engineering Body of Knowledge (Abran et al., 2004).

### 4.2 The artifact development methodology

This research followed a systematic approach in the development of the RA, drawing upon existing methodologies and adapting them to the specific needs of this study. The foundation of this approach was laid by synthesizing key elements from various established RA development methodologies. Notable contributions from Cloutier et al. (2010), Bayer et al. (2004), and Stricker et al. (2010a) were instrumental in forming the basis of the methodology. Each of these studies offered unique perspectives, ranging from contemporary information collection to pattern-based approaches, all contributing to a comprehensive understanding of RA development.

The methodology was further refined by incorporating insights from Galster and Avgeriou (2011) and Nakagawa et al. (2014), who provided a framework for empirically grounded RA development and detailed guidance on RA evaluation. Galster and Avgeriou (2011) have been used as the main artifact development methodology, with the addition of SLRs in the “empirical data acquisition” phase and the Architecture Tradeoff Analyis Method (ATAM) for evaluating the artifact.

Consequently, the methodology adopted for this research was structured into six distinct phases: (1) decision on the type of RA; (2) design strategy; (3) empirical data acquisition; (4) construction of the RA; (5) enabling RA with variability; and (6) RA evaluation.

#### 4.2.1 Step 1: decision on type of the RA

The initial phase in developing the RA involved selecting its type based on the classification framework by Angelov et al. (2009), which categorizes RAs into two main groups: standardization RAs and facilitation RAs. This decision is foundational, guiding the subsequent phases of information collection and RA construction.

The classification framework, based on dimensions of context, goals, and design, was instrumental in identifying the RA type most aligned with the study's objectives. It employs a structured approach using key interrogatives: “When,” “Where,” “Who” for context, “Why” for goals, and “How” and “What” for design.

The chosen RA for this study is a domain-driven distributed BD RA, aiming to support BD system development and promote an effective, scalable data architecture. Therefore, the type is deducted as a standardization RA designed for adaptability across multiple organizational contexts.

#### 4.2.2 Step 2: selection of design strategy

The design strategy for the RA was informed by the frameworks presented by Angelov et al. (2008) and Galster and Avgeriou (2011), which outline two primary approaches: practice-driven (designing RAs from scratch) and research-driven (basing RAs on existing ones). While practice-driven RAs are less common and typically found in nascent domains, research-driven RAs, which amalgamate existing architectures, models, and best practices, are more prevalent in established fields.

Considering these perspectives, this study opts for a research-driven approach. The RA developed leverages existing RAs, concrete architectures, and established best practices. This approach enables the creation of a descriptive design theory that integrates and builds upon the current body of knowledge in the field.

#### 4.2.3 Step 3: empirical acquisition of data

Due to the limitations witnessed by the research method “empirically grounded reference architectures,” specifically the lack of clear guidance on empirical acquisition of data, this phase is augmented by using a SLR on BD RAs presented by Ataei and Litchfield (2022). This SLR is recent and captures the body of knowledge on current RAs in academia and industry.

The findings from this SLR shed light on common components of BD RAs, the limitations of current BD RAs, and various patterns of developing BD RAs.

#### 4.2.4 Step 4: construction of the RA

The construction phase of the RA was informed by the insights and components identified in the studies of Ataei and Litchfield (2022). Moreover, utilizing the ISO/IEC/IEEE 42010 standard (International Organization for Standardization, ISO/IEC) as a foundational guideline, the construction phase was characterized by a selective integration of components.

A key aspect of this phase was the adoption of the Archimate modeling language (Lankhorst, 2013), a component of the ISO/IEC/IEEE 42010 standard. Archimate's service-oriented approach effectively linked the application, business, and technology layers of the RA. This approach is aligned with the concepts proposed by Cloutier et al. (2010) and Stricker et al. (2010b), allowing for a comprehensive understanding of the RA and ensuring its alignment with the study's objectives and context.

#### 4.2.5 Step 5: enabling RA with variability

The integration of variability into the RA is a pivotal aspect, enabling it to adapt to specific organizational regulations and regional policy constraints (Rurua et al., 2019). This adaptability is essential for ensuring the RA's applicability across diverse implementation scenarios.

Variability management is a concept adapted from Business Process Management (BPM) and Software Product Line Engineering (SPLE), fields where managing variations in processes and software artifacts is critical (La Rosa et al., 2009; Rosemann and Van der Aalst, 2007; Hallerbach et al., 2010).

For the RA developed in this study, the mechanism to incorporate variability draws inspiration from the studies of Galster and Avgeriou (2011) and Rurua et al. (2019). This is achieved through the use of Archimate annotations, a method that allows for clear delineation of variability aspects within the RA.

#### 4.2.6 Step 6: evaluation of the RA

The evaluation of the RA is crucial to ensuring it meets its developmental goals, particularly regarding effectiveness and usability (Galster and Avgeriou, 2011). Evaluating an RA involves unique challenges due to its higher abstraction level, diverse stakeholder groups, and focus on architectural qualities (Angelov and Grefen, 2008; Cioroaica et al., 2019; Maier et al., 2013).

Standard ways to evaluate concrete architectures, such as SAAM (Kazman et al., 1994), ALMA (Bengtsson et al., 2004), PASA (Williams and Smith, 2002), and ATAM (Kazman et al., 1998), cannot be directly used for RAs because they need specific stakeholder involvement and scenario-based evaluation, which is hard to do for abstract RAs. This necessitates a customized approach for RA evaluation.

This study adopts a modified evaluation approach, drawing on methodologies adapted for RAs by Angelov et al. (2008) and the extended SAAM approach by Graaf et al. (2005). The process involves creating a prototype of the RA in an actual organizational context, followed by evaluation using ATAM, focussing on aspects such as completeness, buildability, and applicability within the specific context.

This dual approach of theoretical exploration and practical implementation ensures a comprehensive evaluation of the RA. It facilitates understanding the RA's strengths and improvement areas, contributing to its refinement, and enhancing its applicability in various organizational settings (Sharpe et al., 2019; Rohling et al., 2019; Nakagawa et al., 2009).

## 5 Cybermycelium architecture: design and components

This section is composed of the following integral elements: software requirements, design theories, the artifact, and a decision-making aid. First, the requirements that underpin the development of the artifact are explored. Second, the design theories that guide the creation of the artifact are discussed. Finally, the artifact is presented, and its components are described.

### 5.1 Software and system requirements of cybermycelium

As a result of the processes conducted in Section 4.1, a set of requirements for the development of Cybermycelium is identified. These requirements are presented in terms of BD characteristics in Table 1.

**Table 1:** Terramycelium software and system requirements.

**Category**	**Code**	**Requirements**
Volume	Vol-1	Support asynchronous, streaming, and batch processing for data collection from various sources
	Vol-2	Provide scalable storage for massive data sets
Velocity	Vel-1	Support slow, bursty, and high-throughput data transmission between data sources.
	Vel-2	Stream data to consumers in a timely manner
	Vel-3	Ingest multiple, continuous, time-varying data streams
	Vel-4	Support fast search from streaming and processed data with high accuracy and relevancy.
	Vel-5	Process data in real-time or near real-time
Variety	Var-1	Support various data formats: structured, semi-structured, and unstructured
	Var-2	Support aggregation, standardization, and normalization of data from disparate sources
	Var-3	Support adaptation mechanisms for schema evolution
	Var-4	Provide mechanisms to automatically include new data sources
Value	Val-1	Handle compute-intensive analytical processing and machine learning techniques
	Val-2	Support batch and streaming analytical processing
	Val-3	Support different output file formats for different purposes
	Val-4	Support streaming results to consumers
SaP	SaP-1	Protect and retain the privacy and security of sensitive data
	SaP-2	Access control with multi-level, policy-driven authentication on protected data and nodes
Veracity	Ver-1	Support data quality curation: classification, pre-processing, format reduction, and transformation
	Ver-2	Support data provenance: data life cycle management and long-term preservation

### 5.2 The theory

There are various design and kernel theories employed to justify our artifact and the decisions made. These theories are described in the following sub-sections.

#### 5.2.1 A paradigm shift: a distributed domain-driven architecture

Based on the premises discussed in the studies of Ataei and Litchfield (2022), one can infer that the idea of monolithic and centralized data pipelines that are highly coupled and operated by silos of hyper-specialized BD engineers has limitations and can bring organizations into a bottleneck. Therefore, this study explores a domain-driven distributed and decentralized architecture for BD systems and posits that this architecture can address some of the challenges discussed. This idea is inspired by the advancements in software engineering architecture, specifically event-driven microservices architecture (Bellemare, 2020), domain-driven design (Evans and Evans, 2004), and reactive systems (Aceto et al., 2007).

Data usually come in two different flavors: (1) operational data: which serve the needs of an application, facilitate logic, and can include transactional data; and (2) analytical data: which usually have a temporality to it and are aggregated to provide insights. These two different flavors, despite being related, have different characteristics, and trying to lump them together may result in a morass. To this end, Cybermycelium realizes the varying nature between these two planes and respects the difference. Cybermycelium aims to transfigure current architectural approaches by proposing an inversion of control and a topology based on product domains and not technology (Dehghani, 2020). The proposition is that handling two different archetypes of data should not necessarily result in siloed teams, heavy backlogs, and a coupled implementation.

To further elucidate on this matter, we take the example of the microservices architecture. As the industry sailed away from monolithic n-tier architectures into service-oriented architecture (SOA), organizations faced a lot of challenges. One prevalent issue was around the maintenance of the Enterprise Service Bus (ESB) or SOA bus, which is the locus of aggregation. While the aggregation layer could be written very thinly, the reality is that the transformation of XML and logical operations started to bloat the SOA bus. This added a new level of coupling between internal and external elements of the system as a whole (Di Francesco, 2017; Zimmermann, 2017; Waseem et al., 2020).

Microservice architecture, being the evolution of SOA, moved away from smart pipelines into dumb pipelines and smart services, removing the need for the locus of aggregation and control. Moreover, there was no business logic written in the pipelines, and each service was segregated, usually with the help of domain-driven design. Whereas microservices architecture still has its challenges, the gradations of software architectures in the software engineering industry can be analogous to the data engineering domain. One can perceive the pipeline architecture and its coupling nature as similar to SOA and its practice of writing business logic in the SOA bus to connect the services.

Based on the premises discussed and overcoming the limitations, the following underpinning principles for Cybermycelium are posited: (1) distributed domain-driven services with a bounded context; (2) data as a service; (3) data infrastructure automation; (4) governance through a federation service; (5) event-driven services.

#### 5.2.2 Distributed domain-driven services with bounded context

Integral to Cybermycelium is the distribution and decentralization of services into domains that have clear bounded context. Perhaps one of the most challenging things one might face when it comes to architecting a distributed system is: based on what architectural quanta should we break down the system? This issue has been repeatedly discussed, for example, among adopters of microservices architecture. Cybermycelium, inspired by the concept of domain-drive design, tends to store data close to the product domain that relates to it. This implies that data inhere in the product domain and as a facet of it (Laigner et al., 2021).

This is mainly driven by the fact that most organizations today are decomposed based on their products. These products are the capabilities of the business and are segregated into various domains. Domain's bounded context is operated by various teams with different visions and concerns; incorporating data into a bounded context can result in a synergy that can improve the management of evolution and continuous change. This can be micro, such as application developers communicating with data engineers about collecting user data in nested data structures or in flat ones, or macro, such as application developers thinking about redesigning their GraphQL schema in an intermediary layer that may affect the data engineers ingestion services.

The concept of domain-driven design is incorporated into this study to facilitate communication and increase the adoption, rigor, and relevance of the RA. Communication is a key component of any software development endeavor (Sudhakar, 2012), and without it, essential knowledge sharing can be compromised. Often, data engineers and business stakeholders have no direct interaction with one another. Instead, domain knowledge is translated through intermediaries such as business analysts or project managers to a series of tasks to be done (Khononov, 2021). This implies at least two translations from two different ontologies.

In each translation, information is lost, which is essential domain knowledge, and this implies risk to the overall data quality. In such a data engineering process, the requirements often get distorted, and the data engineer has no awareness of the actual business domain or the problem being addressed. Often times, problems being solved through data engineering are not simple mathematical problems or riddles but rather have broader scopes. An organization may decide to optimize workflows and processes through continuous data-driven decision-making, and a data architecture that is overly centralized and not flexible can risk project failure.

To address this challenge, domain-driven design proposes a better approach to conveying knowledge from domain experts to data engineers. In domain-driven design, instead of intermediary translations, business domains are projected into actual data engineering, emphasizing the creation of one shared terminology, which is the “ubiquitous language.” This study does not aim to explore all facets of domain-driven design, but it is worth mentioning that each business has its own domain and constituent core, generic, and supporting sub-domains, and this varies from context to context.

#### 5.2.3 Data as a service

Data can be conceived as the fourth dimension of a product, next to UI/UX, business, and application. Each domain provides its data as a service. These data consist of both operational and analytical data. This also implies that any friction and coupling between data are removed. For instance, the “invoice” domain will provide transactional data about the number of invoices and total of discounts, along with analytical data such as which practices have created what number of invoices in what period of time.

However, this data-as-a-service model should be carefully implemented to account for explorability, discoverability, security, and quality. The data provided as a service should have the identical qualities as customer-facing products. This also implies that a product owner should now treat the data facet as an aspect of the product and employ objective measures that assure the desired quality. These measures can include net promoter scores from data consumers, data provenance, and decreased lead time. Product owners, in addition to the application and design aspects of the product, must now incorporate this new facet and try to understand the needs of data consumers, how they consume the data, and what the common tools and technologies are to consume the data. This knowledge can help shape better interfaces for the product.

Product domains may also need to ingest data from upstream domains, and this requires the definition of clear interfaces. Furthermore, each domain should also account for metadata. Metadata is derived from the nature of the product and its data lifecycle. Data can be ingested and served in various forms, such as tables, graphs, JSON, Parquet, events, and many more, but in order for the data to be useful for analytical purposes, there is a need to associate the data with its corresponding metadata that encompasses semantics and history.

#### 5.2.4 Data infrastructure automation

As the number of product domains increases, the effort required to build, deploy, execute, and monitor services increases. This includes the data pipelines required for that product domain to carry out its functions. The platform skills required for this kind of study are usually found in Devops engineers and site reliability engineers. Application developers and data engineers are usually not adept at carrying out such workloads in an efficient manner. For this reason, there is a need for highly abstract, reusable infrastructural components that can be easily utilized. This implies that teams should be equipped with the required infrastructure as a service that can be easily employed to account for BD needs.

One way to provision such infrastructure as a service is to utilize Infrastructure as a Code (IaaS) software tools such as Terraform (Hashicorp, 2022) and follow the principles of GitOps. In addition, data infrastructure may be extended based on the currently running infrastructure for application payloads. However, this might be challenging as the BD ecosystem is growing rapidly. While a software application might be running on an EC2 worker node in an EKS cluster on Amazon, the BD system may be running on a Databricks cluster or using a customer data platform (CDP) solution such as Segment (Segment, 2022).

Nevertheless, this should not be a daunting task, as one can simply extend the EKS configs and add a new pod to the network, which instals Databricks through a Helm Chart (Helm, 2022). In addition, the data infrastructure should be accompanied by proper tooling.

A mature infrastructure as a service should provide the team with core infrastructures such as BD storage, stream processing services, batch processing services, event backbones, message queues, and data integration technologies. Composing data and application infrastructure together provides a coherent, cost-efficient, and interoperable infrastructure.

#### 5.2.5 Governance through a federation service

The other principle of Cybermycelium is global governance, or the global standardization of services. This principle is perhaps a lesson learnt from the studied application of microservices architecture in the industry (Alshuqayran et al., 2016). Distributed architectures are made up of independent collections of nodes with distinct lifecycles that are deployed separately and are owned by various teams. As the number of these services grows and the interconnections increase, the challenge of maintaining and scaling the system increases. This means services need to interoperate, ingest data from other services, perform graph or set operations in a timely manner, and do stream processing.

To scale and maintain these independently deployed yet interconnected services, Cybermycelium needs a governance model that embraces domain autonomy, decentralization, automation, Devops, and interoperability through federated government. This requires a shift in thinking, which obsoletes many prevalent assumptions about software and data engineering. The point of federation is not to suppress or kill the creativity and innovation of the teams but rather to introduce global contracts and standards that are in line with the company's resources and vision. Nevertheless, finding equilibrium between the right amount of centralisation and decentralization presents a challenge. For instance, semantic-related metadata can be left to the product domain to decide, whereas policies and standards for metadata collection should be global. This is somewhat analogous to architectural principles in TOGAF's ADM (Josey, 2016).

The definition of these standards is up to the architecture, or architectural governance group, and is usually achieved through service level objectives (SLOs) or well-defined contracts and standards.

#### 5.2.6 Event-driven services

Cybermycelium has been designed in a decentralized and distributed manner. Despite the advantages of decentralized systems in terms of maintenance and scalability, communication between the services remains a challenge Ataei and Staegemann (2023). As the number of services grows, the number of communication channels increases, and this soon turns into a nexus of interconnected services that each try to meet its own end. Each service will need to learn about the other services, their interfaces, and how the messages will be processed. This increases the coupling between services and makes maintenance a challenging task. It is argued that this should not be the aim of a distributed RA such as Cybermycelium.

One approach to alleviating these issues is asynchronous communication between services through events. This is a different paradigm from a typical REST style of communication. A point-to-point communication occurs between services as a series of “commands,” like getting or updating certain resources, whereas event-driven communication happens as a series of events. This implies that instead of service A commanding service B for certain computations, service B reacts to a change of state through an event without needing to know about service A.

This provides a *dispatch and forget* kind of model in which a service is only responsible for dispatching an event to a topic of interest for the desired computation. In this way, the service does not need to wait for the response and see what happens after the event is dispatched and is only responsible for dispatching events through a well-defined contract. Underlying this paradigm, services do not need to know about each other, but rather they need to know what topic they are interested in.

This is analogous to a restaurant, where instead of a waiter needing to communicate directly with another waiter, the chef, and the cook, they all react to certain events, such as customers coming in or an order slip being left on the counter. The subtlety lies in the underlying paradigm and philosophy of *event* instead of *command*. This paradigm solves many issues of communication in distributed BD systems, such as long running blocking tasks, throughput, maintenance, scale, and the ripple effect of service failure.

In Cybermycelium, eventual consistency (BASE) is preferred over ACID transactions for performance and scalability reasons (Xie et al., 2014). The details of these two varying kinds of transactions are outside the scope of this study.

### 5.3 The artifact

**Figure 1:**
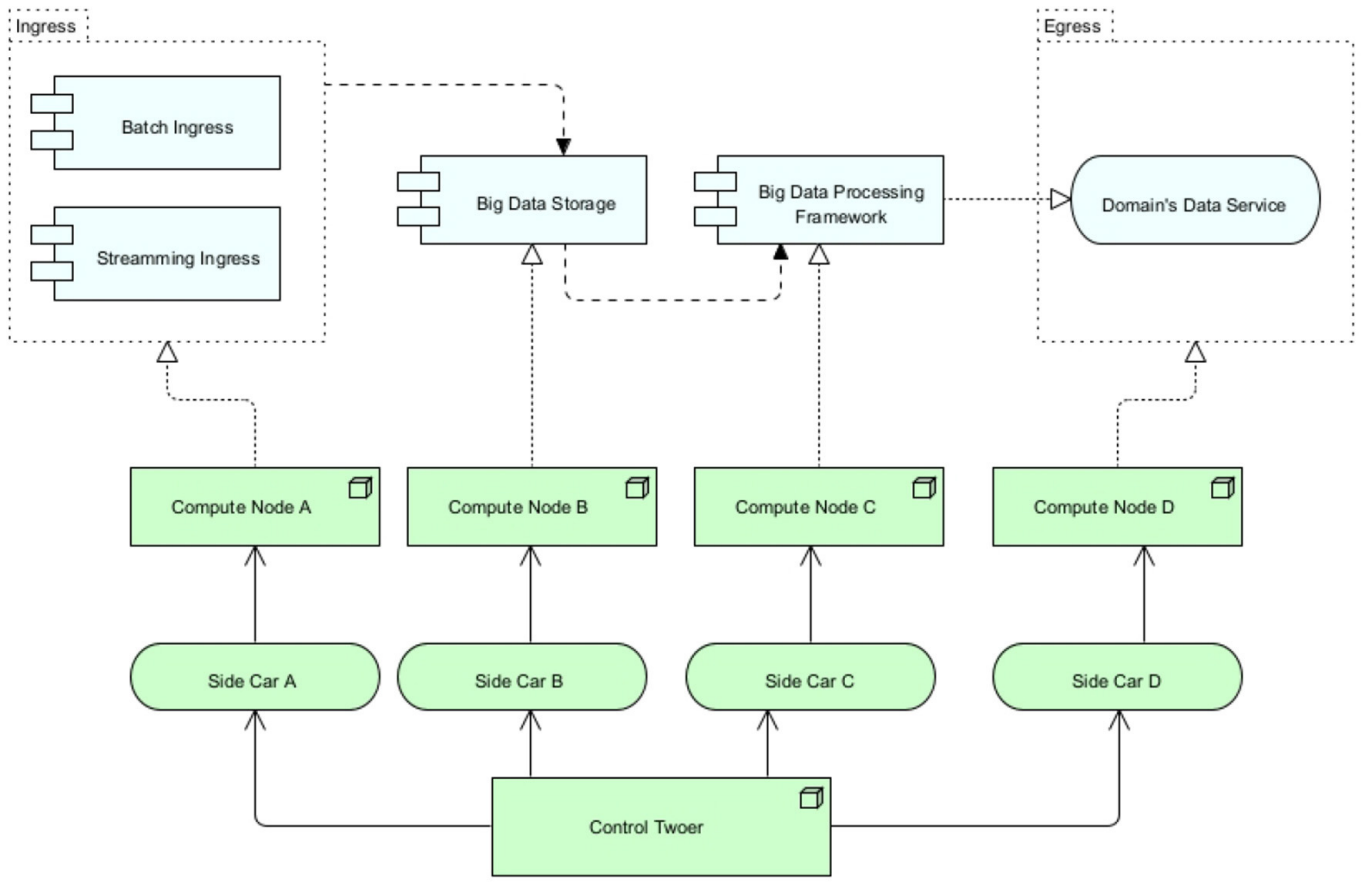
Cybermycelium product domain design.

**Figure 2:**
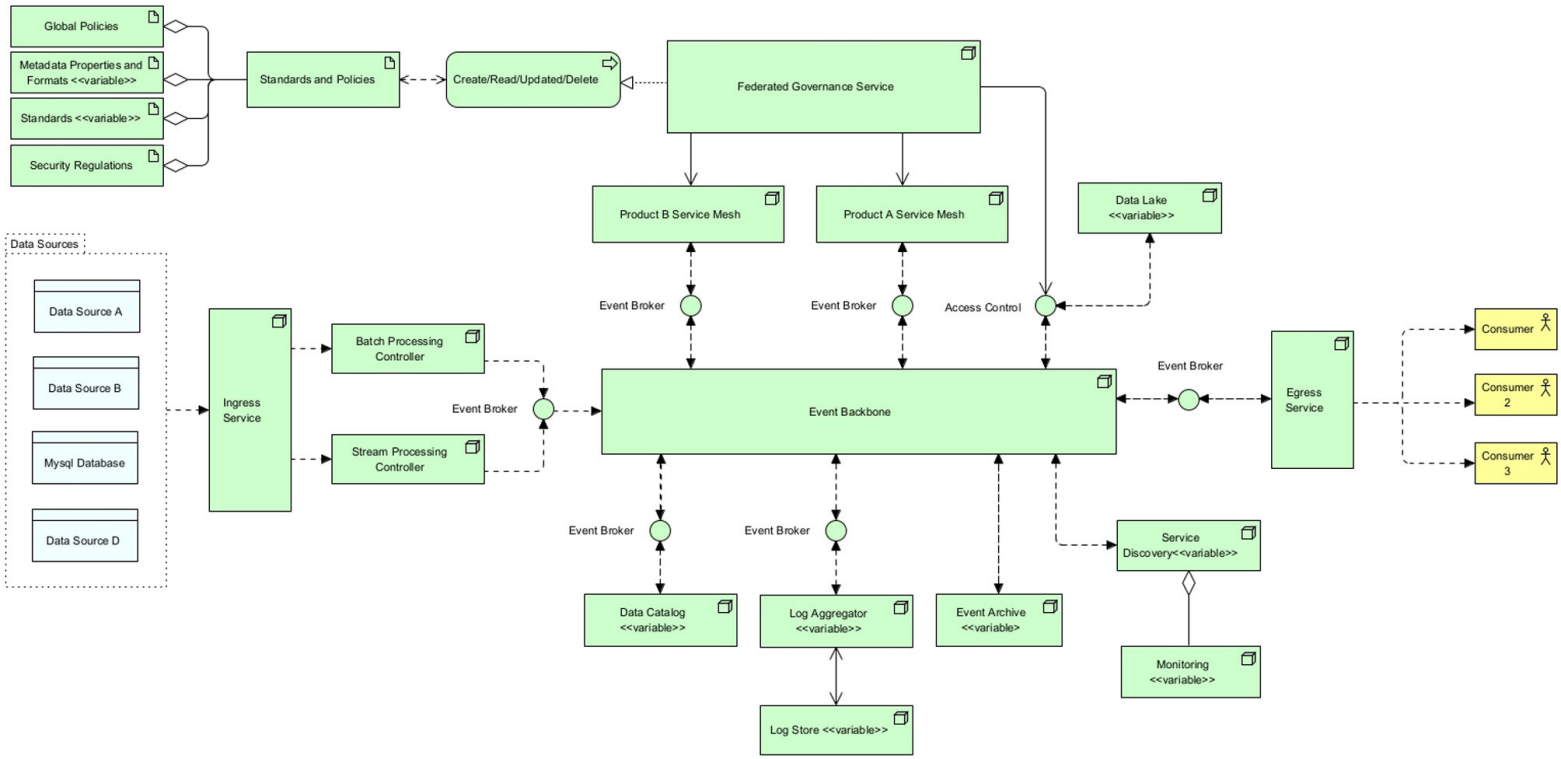
Cybermycelium Big Data Reference Architecture.

After having discussed many kernel and design theories, the necessary theoretical foundation is created for the design and development of the artifact. Cybermycelium is created with Archimate and displays the RA mostly in the technology layer. Displaying these services in the technology layer means that it is up to the designer to decide what flow and application should exist at each node. For the sake of completion, and as every piece of software is designed to account for a business need, a very simple BD business process is assumed. While this business layer could vary in different contexts, Cybermycelium should be able to have the elasticity required to account for various business models. This artifact is delineated in Figure 2. To ease understanding of the RA, the product domain is sub-diagrammed in Figure 1.

#### 5.3.1 Implementation guide

A series of template scripts and configurations for system instantiation are hosted in an external repository, providing access to the most recent versions. These materials encompass common setup scenarios and infrastructure for components within the Cybermycelium architecture. This repo aims to provide a skeleton of the project and some of its major components. The developer or the architect can choose to change Docker images, choose different Helm charts, and deploy new OLAP or OLTP systems. This repo can serve as a starter example and is available at Ataei (2023).

#### 5.3.2 Components

Cybermycelium is made up of eight main components and seven variable components, as depicted in Figure 2. For simplicity, sub-components are counted as just components. For example, *Global Policies* component is a sub-component of *Standards and Policies* component, but it is counted as one non-variable component. To help with transparency, recreatability, and generalisability, explanations of some components are accompanied by an implementation guide and variable component guidance.

These elements are the following:

1. Ingress service: the ingress service is responsible for exposing the necessary port and endpoint for the data to flow to the system. Depending on the nature of the request, the ingress service will load balance to either a batch processing controller or a stream processing controller. It is essential for the ingress service to operate asynchronously to avoid any potential choke points. In addition, ingress handles the SSL termination and potentially name-based virtual hosting. Ingress has several benefits. First, it helps with security by preventing port proliferation and direct access to services. Second, it helps with performance by distributing requests based on their nature and SSL termination. Third, if there is a need for object mutation through a proxy, ingress is the best architectural construct. Having an ingress also means that the point of entry is clear, which makes monitoring easier and allows for other components of the architecture to remain in private networks. This component addresses the requirements Vol-1, Vol-2, Var-1, Var-3, Var-4, Val-1, Val-3, Val-4, SaP-1, and SaP-2.
Implementation guide: for high data throughput or handling sensitive data, it is recommended to employ robust security measures such as SSL/TLS encryption for data in transit, firewall configurations, and rate limiting to protect against distributed denial-of-service (DDoS) attacks. Configuration should adhere to industry best practices for network security and data protection. In smaller-scale implementations or environments with lower security risks, a simplified ingress setup may suffice. This entails configuring the necessary ports and endpoints with appropriate network policies and employing basic SSL termination. Monitoring and logging should be enabled to track ingress service performance and security incidents. A simple and advanced example of an ingress for Kubernetes can be found in the templates folder at Ataei (2023). The configurations provided in the repository are only for educational purposes and are not production ready.
2. Batch processing controller: the batch processing controller is responsible for dispatching batch events to the event backbone. This service should be a small one (it could be a Lambda) with the main responsibility of receiving a request for batch processing and dispatching an event to the event broker. Because the nature of the request is of type batch and has been clearly distinguished by the ingress, the batch processing controller can dispatch events in bulk and asynchronously. This is the main difference between this service and a stream processing controller. The batch processing controller can execute other non-compute-intensive tasks, such as scrubbing properties from the given data or adding headers. Having a specific controller for batch processing improves monitoring and allows for customized batch event production. This component addresses the requirements for Vel-1, Val-1, and Val-2.
Implementation considerations for batch processing controller: when implementing the batch processing controller, scalability is a primary concern. The service should scale to handle varying volumes of data, possibly through containerisation strategies or serverless architectures such as AWS Lambda. Error handling and retry mechanisms are crucial to managing failed batch jobs effectively. Integrating comprehensive monitoring and alerting is essential to tracking job status, performance metrics, and system health. Maintaining data consistency and integrity throughout the processing lifecycle is imperative for ensuring reliable operations.
3. Stream processing controller: the stream processing controller is responsible for dispatching streaming events to the event backbone through the event broker. This service has been segregated from the batch service as it has to account for a different nature of events. Streams are synchronous in nature and can require high throughput. This service is a small one as well, but non-heavy computations such as enabling stream provenance and one-pass algorithms can be utilized. Having a specific controller for stream processing means that custom attributes can be associated with stream events, and the events can potentially be treated differently based on the nature of the system. This also eases monitoring and discovery. This component addresses the requirements Vol-1, Vel-1, Vel-2, Vel-4, Vel-5, and Val-2.
Implementation considerations for stream processing controller: the batch processing controller is vital for handling large, non-time-sensitive data volumes. However, in environments where real-time or near-real-time data processing is paramount, the emphasis might shift toward stream processing controllers. These controllers are optimized for handling continuous data streams, providing timely insights and responses, and are particularly beneficial in scenarios such as real-time analytics, online transaction processing, or monitoring systems.
For environments dominated by real-time data needs, transitioning to stream processing involves utilizing technologies like Apache Kafka or Amazon Kinesis, designed for high-throughput, low-latency processing. Sometimes, combining micro-batching with stream processing can balance the need for real-time processing with the efficiencies of batch processing. Effective state management is critical across distributed components in real-time processing systems. Optimizing the entire pipeline for low latency, from data ingestion to processing and eventual action or storage, is essential.
Whether opting for batch or stream processing or a hybrid approach, the architecture should align with the specific data, latency, and processing requirements of the application or system. The decision should consider the balance between immediate data handling needs and the efficiencies of batch operations, ensuring that the system is both responsive and efficient.
4. Event broker: event brokers are designed to achieve “inversion of control.” As the company evolves and requirements emerge, the number of nodes or services increases, new regions of operations may be added, and new events might need to be dispatched. As each service has to communicate with the rest through the event backbone, each service will be required to implement its own event handling module. This can easily turn into a spaghetti of incompatible implementations by various teams and can even cause bugs and unexpected behaviors. To overcome this challenge, an event broker is introduced to each service of the architecture. Each service connects to its local event broker and publishes and subscribes to events through that broker. One of the key success criteria of the event broker is a unified interface that sits at the right level of abstraction to account for all services in the architecture. Event brokers, being environmentally agnostic, can be deployed on any on-premise, private, or public infrastructure. This frees up engineers from having to think about the event interface they have to implement and how it should behave. Event brokers can also account for more dynamism by learning which events should be routed to which consumer applications. Moreover, event brokers do also implement circuit breaking, which means if the service they have to broke to is not available and does not respond for a certain amount of time, the broker establishes the unavailability of the service to the rest of the services, so no further requests come through. This is essential to preventing a ripple effect over the whole system if one system fails. This component indirectly addresses the requirements: Val-1, and Ver-1.
5. Event backbone: this is the heart of the Cybermycelium, facilitating communication among all the nodes. The event backbone in itself should be distributed and ideally clustered to account for the ever-increasing scale of the system. Communication occurs as choreographed events from services analogous to a dance troupe. In a dance troupe, the members respond to the rhythm of the music by moving according to their specific roles. Here, each service (dancer) listens and reacts to the event backbone (music) and takes the required action. This means services are only responsible for dispatching events in a ‘dispatch and forget' model and subscribe to the topics that are necessary to achieve their ends. The event backbone thus ensures a continuous flow of data among services so that all systems are in the correct state at all times. The event backbone can be used to mix several streams of events, cache events, archive events, and other manipulations of events, so long as it is not too smart or does not become an ESB of SOA architectures. Ideally, an architect should perceive the event backbone as a series of coherent nodes that aim to handle various topics of interest. Over time, the event backbone can be monitored for access patterns and tuned to facilitate communication in an efficient manner. This component addresses the requirements Vel-1, Vel-2, Vel-3, Vel-4, Vel-5, Val-1, Val-2, Ver-1, Ver-2, and Ver-3.
Implementation considerations for event backbone: when implementing the event backbone, scalability and fault tolerance are paramount. The backbone should be designed to handle high-throughput event streams with low latency. Consider using a distributed streaming platform such as Apache Kafka or Apache Pulsar, which provide strong durability guarantees and support for exactly-once processing semantics. Implement a partitioning strategy that allows for parallel processing and ensures event ordering within partitions. State management is crucial; use a robust state backend (like RocksDB) to handle large state sizes and enable efficient checkpointing for fault tolerance. Implement backpressure mechanisms to handle scenarios where event production outpaces consumption. Careful configuration of event time and watermarks is essential for handling out-of-order events and late data accurately.
6. Egress service: the egress service is responsible for providing the necessary APIs for the consumers of the system to request data on demand. This is a self-serve data model in which data scientists or business analysts can readily request data from various domains based on the data catalog. Clients can first request a data catalog and then use the catalog to request the product domain that accounts for the desired data. This request can include several data products. Egress is responsible for routing the request to the data catalog and to the corresponding product “service mesh” to resolve values. The egress realizes the address for service meshes and other services through the data catalog and service discovery. The egress service should cache the resolved addresses and values to increase performance and response time. An architect can even choose to implement a complete query cache component inside the egress service; however, that will increase complexity and can affect modifiability. This component is to avoid having people request data directly from data engineers for various BD requirements and means that people can just request what data they need, analogous to a person who orders food at a restaurant, with the menu being the data catalog and egress being the waiter. This component addresses the requirements for Vel-2, Vel-4, Val-3, Val-4, SaP-1, and SaP-2.
7. Product domain service mesh: as previously discussed, a product is a capability of the business, and each product has its own domain consisting of the bounded context and the ubiquitous language. From a system and architectural point of view, these domains are referred to as a “service mesh” (Figure 1). Each service mesh is made up of a batch ingress, stream ingress, BD storage, BD processing framework, domain's data service, the required compute nodes to run these services, a sidecar per service, and a control tower. These components provide the necessary means for the domain to achieve its ends. This architectural component removes the coupling between the teams and promotes team autonomy. This means people across various teams are enhanced with the desired computational nodes and tools necessary and can operate with autonomy and scale without having to be negatively affected by other teams or having friction with platform teams or siloed data engineering teams. Depending on the context and the business, the architect may create several domains. This component indirectly addresses Vol-1, Vel-3, Vel-4, Vel-5, Var-1, Var-2, Var-3, Val-1, Val-2, Val-3, Val-4, Sap-1, SaP-2, Ver-1, Ver-2, and Ver-3.
Implementation considerations for product domain service mesh: when implementing the product domain service mesh, focus on enhancing observability, security, and traffic management within and between product domains. Utilize a service mesh implementation such as Istio, which can be deployed on Kubernetes. Implement sidecar proxies (such as Envoy) alongside each service to handle inter-service communication, enabling features such as load balancing, circuit breaking, and telemetry collection without modifying application code. Configure mutual TLS (mTLS) between services for secure communication. Implement fine-grained access controls using Istio's authorization policies. Utilize Istio's traffic management features for canary deployments and A/B testing within product domains. Implement distributed tracing to monitor request flows across services, aiding in performance optimization and debugging.
8. Federated governance service: evidently, Cybermycelium is a distributed architecture that encompasses a variety of independent services with independent lifecycles that are built and deployed by independent teams. Whereas teams have their autonomy established, to avoid haphazard, out-of-control, and conflicting relations, there should be a global federated governance that aims to standardize these services. This will facilitate the interoperability between services, communication, and aggregates and even allow for a smoother exchange of members across teams. This also means the most experienced people at a company, such as technical leads and lead architects, will prevent potential pitfalls that more novice engineers may fall into. However, the aim of this service is not to centralize control in any way as that would be going a step backward into the data warehouse era. The aim of this service is to allow autonomous flow in the river of standards and policies that tend to protect the company from external harm. For instance, failing to comply with GDPR while operating in Europe can result in fines of up to 10 million euros, and this may not be something that novice data engineers or application developers are fully aware of. The real challenge for the governance team is then to figure out the necessary abstraction of the standards for the governance layer and the level of autonomy given to the teams. The federated governance service is made up of various components, such as global policies, metadata elements and formats, standards, and security regulations. These components are briefly discussed below:
(a) Global policies: general policy governs the organizational practice. Both internal and external factors could have an impact on this. For instance, complying with GDPR could be a company's policy and should be governed through the federated governance service.
(b) Metadata properties and formats: this is an overarching metadata standard defining the required elements that should be captured as metadata by any service within the organization; it can also include the shape of metadata and the properties of it. For instance, the governance team may decide that each geographical metadata should conform to ISO 19115-1 (ISO, 2019).
Variable components guidance: in the event of deploying an internal application where the data are transient and not subjected to compliance scrutiny, the complexity of the metadata can be significantly reduced or omitted. An architect may streamline metadata to include essential elements that support basic operational requirements, foregoing expansive metadata schemes typically necessitated by external regulatory bodies.
(c) Standards: overall standards for APIs (for instance, Open API), versioning (for instance, SemVer), interpolation, documentation (for instance, Swagger), data formats, languages supported, tools supported, technologies that are accepted, and others.
Variable components guidance: in scenarios where a system operates in isolation from external interfaces or in a highly specialized domain with unique requirements, adherence to common standards may be relaxed or omitted. The architect must ensure that any deviation from established standards does not impede future integration efforts or system scalability. The decision to omit standardization should be deliberate, with a focus on maintaining system agility while safeguarding against potential technical debt.
(d) Security regulations: company wide regulations on what is considered secured, what software is allowed, how interfaces should be conducted, and how the data should be secured. For instance, a company may choose to alleviate the risks associated with OWASP's top 10 application security risks.
While the abovementioned components are promoted as the bare minimum, an architect may decide to omit or add a few more components to the federated governance service. This component can indirectly affect all requirements.
9. Data catalog (variable): as the number of products increases, more data become available to be served to consumers, interoperability increases, and maintenance becomes more challenging. If there is no automatic way for various teams to have access to the data they desire, a rather coupled and slow BD culture will evolve. To avoid these challenges and to increase discoverability, collaboration, and guided navigation, the service data catalog should be implemented. The data catalog is listed as a must-have by Gartner (Ehtisham Zaidi, 2019) and introduces better communication dynamics, easier data serve by services, and intelligent collaboration between services. This component addresses the requirements Vel-4, Var-1, Var-3, and Var-4.
Variable component guidance: in scenarios where the organization utilizes a single or limited number of data sources, the structure of the data catalog could be condensed or entirely omitted. The architect could pivot toward a direct query approach against the source systems, especially in environments where data lineage and sourcing are not of paramount concern.
10. Logging aggregator and log store (variable): if all services employ the idea of localized logging and simply generate and store logs in their own respective environments, debugging, issue-finding, and maintenance can become challenging tasks. This is due to the distributed nature of Cybermycelium and the requirements to trace transactions among several services. To overcome this challenge, the log aggregator pattern popularized by Chris Richardson is employed (Richardson, 2018). The log aggregator service is responsible for retrieving logging events through the event broker from individual services and writing the collected data into the log store. The log aggregator configuration and semantics are up to the designer and architecture team. This allows for the distributed tracing and graceful scaling of organizational logging strategies. This component indirectly addresses the requirements Vol-1, Vel-1, Val-1, and Ver-1.
Variable components guidance: for applications with a narrow scope of operation and minimal user base, such as a prototype or an internally used tool, the logging aggregator may be omitted. In such cases, direct log analysis methods may suffice, freeing the system from the additional layer of log aggregation complexity.
11. Event archive (variable): as the quantity of services grows, the topics in the event backbone increase, and the number of events surges. Along the lines of these events, there could be a failure, resulting in a timeout and a loss of a series of events. This puts the system in the wrong state and can have detrimental ripple effects on all services. Cybermycelium tends to handle these failures by using an event archive. The event archive, as the name states, is responsible for registering events, so they can be retrieved in the event of failure. If there was a blackout in a certain geographical location and the event backbone went down, the backbone could recover itself and bring back the right state of the system by reading the events from the event archive. The event broker is responsible for circuit breaking, so services do not request any more events to the backbone while it is down. The time to expire and what events should be archived are decided based on the context in which Cybermycelium is implemented. This component indirectly addresses the requirements Vol-1, Vel-1, Val-1, and Ver-1.
Variable components guidance: in a development or testing environment where events are non-critical, the event archive could be omitted. The architect might determine that the operational overhead of maintaining an archival system outweighs its benefits in a non-production scenario.
12. Data lake (variable): whereas Cybermycelium is a great advocate of decentralized and distributed systems, it is not necessary for each product domain to have its own kind of data lake or data storage. This is to prevent duplication, contrasting data storage approaches, decreased operability among services, and a lack of unified raw data storage mechanisms. A data lake has been designed to store large volumes of data in raw format before it can be accessed for analytics and other purposes. This means data can be first stored in the data lake with corresponding domain ownership before it needs to be accessed and consumed by various services. Structured, semi-structured, unstructured, and pseudo-structured data can be stored in the data lake before it gets retrieved for batch and stream processing. Nevertheless, this does not imply that all data should directly go to the data lake; the flow of data is determined based on the particularities of the context in which the system is embodied. A suitable approach is to assign each team ownership of a specific storage unit within the data lake, managed through access control. This component addresses the requirements Vol-2, Vel-1, Var-1, Var-3, Var-4, and Val-3.
Variable component guidance: a scenario that warrants the omission of complex partitioning strategies within the data lake is when the enterprise operates on a small-scale data footprint with homogeneous data types. Here, an architect may favor a simplified, flat-storage approach, eliminating the need for elaborate partitioning and the overhead it entails.
13. Service discovery (variable): in a distributed setup such as Cybermycelium, how do services discover the location of other services? This is achieved through service discovery. As the practice of hard-coding service addresses in configuration files is not a maintainable or scalable approach, one has to think about an automated, scalable solution in which services can become discoverable by other services. The service discovery node is responsible for this job. This is achieved through services registering themselves with the service discovery node when they boot up. Service discovery then ensures that it keeps an accurate list of services in the system and provides the API necessary for others to learn about the services. For instance, it is idiomatic for an engineer to specify a command to be executed when a Docker container starts (*Node server.js*); thus, one can imagine extending the boot up instructions to achieve registration to the service discovery node. This somewhat resembles DHCPs and house wifi networks. This component indirectly addresses the requirements Vel-2, Vel-4, Var-2, Var-4, Val-3, Val-4, and SaP-2.
Variable component guidance: for monolithic applications or when services are statically assigned and do not require discovery for communication, the service discovery component can be omitted. An architect might bypass this component to reduce architectural complexity in a stable and predictable deployment environment.
14. Monitoring (variable): monitoring systems are integral to the robustness of a highly dynamic ecosystem of distributed systems and directly affect metrics such as mean time to resolution (MTTR). Services emit large amounts of multi dimensional telemetry data that cover a vast spectrum of platform and operating system metrics. Having this telemetry data captured, handled, and visualized helps systems engineers, software reliability engineers, and architects proactively address upcoming issues. Based on these premises, the main responsibility of this service is to capture and provide telemetry data from other services to increase the overall awareness of the Cybermycelium ecosystem. This service is tightly aggregated with the service discovery. Monitoring services help store this data to fuel proactive actions. This component indirectly addresses all requirements.
Variable component guidance: in smaller, less complex systems where the operational state can be ascertained without extensive monitoring, an architect may decide to omit advanced monitoring configurations. This could apply to single-service applications or ones with minimal integration points, where basic monitoring suffices.

#### 5.3.3 Variable components

The variable elements in Cybermycelium can be adjusted, modified, or even omitted based on the architect's decision and the particularities of the context. The aim of this RA is not to limit the creativity of data architects but to facilitate their decision-making process through the introduction of well-known patterns and best practices from different schools of thought. While it is still recommended to keep the variable components, an architect may decide to embark on a more complicated metadata approach rather than just a data catalog. For brevity, this study does not elaborate on all the alternative options for each variable module. This is due to the fact that industry constantly changes, and architects constantly aim to design systems that address emerging problem domains.

### 5.4 Decision-making aid

**Figure 3:**
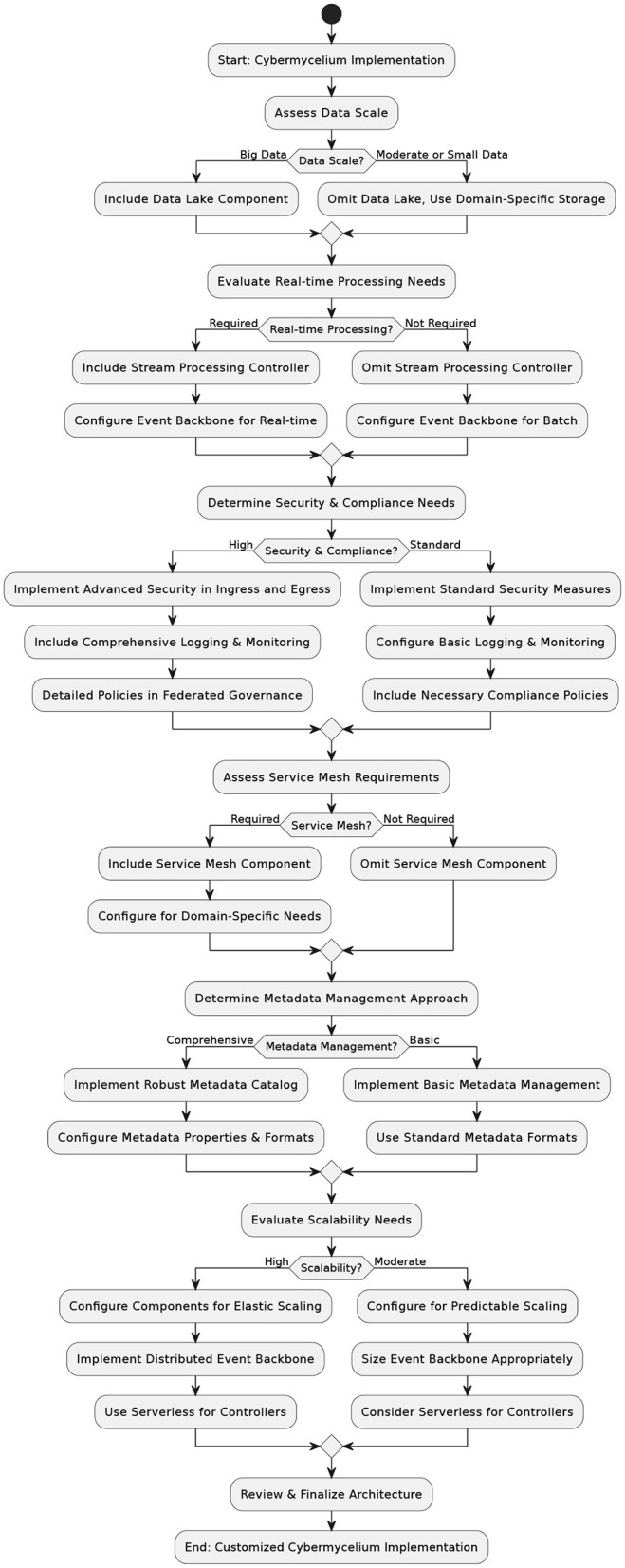
Decision-making tree for Cybermycelium.

The component decision tree illustrated in Figure 3 is designed to guide architects through the intricate process of selecting, modifying, or omitting various components based on a multitude of factors. This tool addresses architectural flexibility and varying application scenarios, providing a structured pathway to informed architectural choices.

Developed through an analysis of the architecture's components and influenced by factors such as data volume, system complexity, and compliance needs, the tree presents decision nodes leading to multiple pathways. These nodes represent key architectural considerations, directing the implementation toward a configuration that aligns with organizational objectives and technical requirements. Architects begin with an assessment of the organizational context, which influences the direction and complexity of subsequent decisions. As they traverse the tree, they engage with decisions concerning data scale, processing needs, security, compliance, and scalability, each with its ramifications and trade-offs.

## 6 Evaluation

Of particular importance to the development of an RA is its evaluation. As previously discussed, the aim is to evaluate the RA's correctness and utility by assessing its transformation into an effective, context-specific concrete architecture, following the guidelines of ATAM. The main goal of ATAM is to appraise the consequences of architectural decisions in the light of quality attributes. This method ensures that the architecture is on the right trajectory and in line with the context. By uncovering key architectural trade-offs, risks, and sensitivity points, ATAM analysis increases confidence in the overall design.

For ATAM to be successful, there should not be a precise mathematical analysis of the system's quality attributes, but rather trends should be identified where architectural patterns are correlated with a quality attribute of interest. For brevity purposes, ATAM is not expanded on, nor are the details of each step in it. Only an explanation of how the evaluation has been conducted through ATAM is provided. It is important to note that this was not a setup in which an outside evaluation team would come to a company to evaluate an architecture in practice, but it was artifact of this study that was brought into a company to test its utility and relevance.

While this could have been achieved with technical action research or lightweight architecture evaluation, ATAM was found to be in line with the conceptual constructs, which are architectural constructs. ATAM provided us with a framework to discuss architectural concepts in a rigorous way (Wieringa, 2014). A prototype was created and evaluated internally, introducing potential bias. To avoid bias, a third-party researcher, familiar with ATAM, was invited to observe the overall process and partake in architectural probing questions.

For instantiation of the RA, the ISO/IEC 25000 SQuaRE standard (Software Product Quality Requirements and Evaluation) (ISO, 2014) has been utilized for technology selection. Notwithstanding, this standard was not fully adopted. The technology research phase combined a structured literature review with hands-on exploratory testing, ensuring a comprehensive understanding of potential technologies for the Cybermycelium architecture. The literature review delved into academic papers, industry reports, product documentation, and user testimonials to understand various technologies, theoretical underpinnings, practical applications, strengths, and limitations. This phase was essential in assessing the alignment of each technology with the specific requirements of Cybermycelium.

Concurrently, exploratory testing provided firsthand insights into the functionalities, maintainability, compatibility, and portability of the technologies. This practical examination ensured that each technology was rigorously assessed against established evaluation criteria, with findings documented for subsequent analysis. For example, when selecting a distributed streaming platform for the event backbone, Apache Kafka, Amazon Kinesis, and Azure Event Hubs were evaluated. The literature review revealed that Apache Kafka had a strong academic foundation, extensive industry adoption, and a wide range of connectors and integrations.

Exploratory testing confirmed Kafka's high throughput, low latency, and scalability. Moreover, Kafka had over 27 thousand stars on Github with over 11,000 issues closed, portraying the maturity and reliability of the library. The evaluation matrix scored Kafka highly in terms of functional suitability, reliability, and maintainability. As a result, Apache Kafka was chosen as the technology for the event backbone in the Cybermycelium instantiation.

Consequently, popular open-source tools that support the architectural requirements of Cybermycelium have been chosen. Developing tools from scratch was not favored, as that would delay the evaluation artifact and this would affect the stakeholders negatively. In addition, many mature tools exist that satisfy the architectural requirements of Cybermycelium, so therefore “reinventing the wheel” was unnecessary. Table 2 is the breakdown of how each technology was implemented in the Cybermycelium prototype:

**Table 2:** Components, technologies, and requirements in the Cybermycelium evaluation.

**Component**	**Technology**	**Requirements addressed**
Ingress service	Nginx	Vol-1, Vol-2, Var-1, Var-3, Var-4, Val-1, Val-3, Val-4, SaP-1, SaP-2
Batch processing controller	AWS lambdas	Vel-1, Val-1, Val-2
Stream processing controller	AWS lambdas, kafka streams	Vol-1, Vel-1, Vel-2, Vel-4, Vel-5, Val-2
Event broker	Kafka broker	Val-1, Ver-1 (indirectly)
Event backbone	Kafka	Vel-1, Vel-2, Vel-3, Vel-4, Vel-5, Val-1, Val-2, Ver-1, Ver-2, Ver-3
Egress service	AWS application load balancer, node JS	Vel-2, Vel-4, Val-3, Val-4, SaP-1, SaP-2
Product domain service mesh	Istio, envoy	Vol-1, Vel-3, Vel-4, Vel-5, Var-1, Var-2, Var-3, Val-1, Val-2, Val-3, Val-4, SaP-1, SaP-2, Ver-1, Ver-2, Ver-3
Data lake	AWS S3	Vol-2, Vel-1, Var-1, Var-3, Var-4, Val-3
Event archive	AWS S3	Vol-1, Vel-1, Val-1, Ver-1 (indirectly)

**Figure 4:**
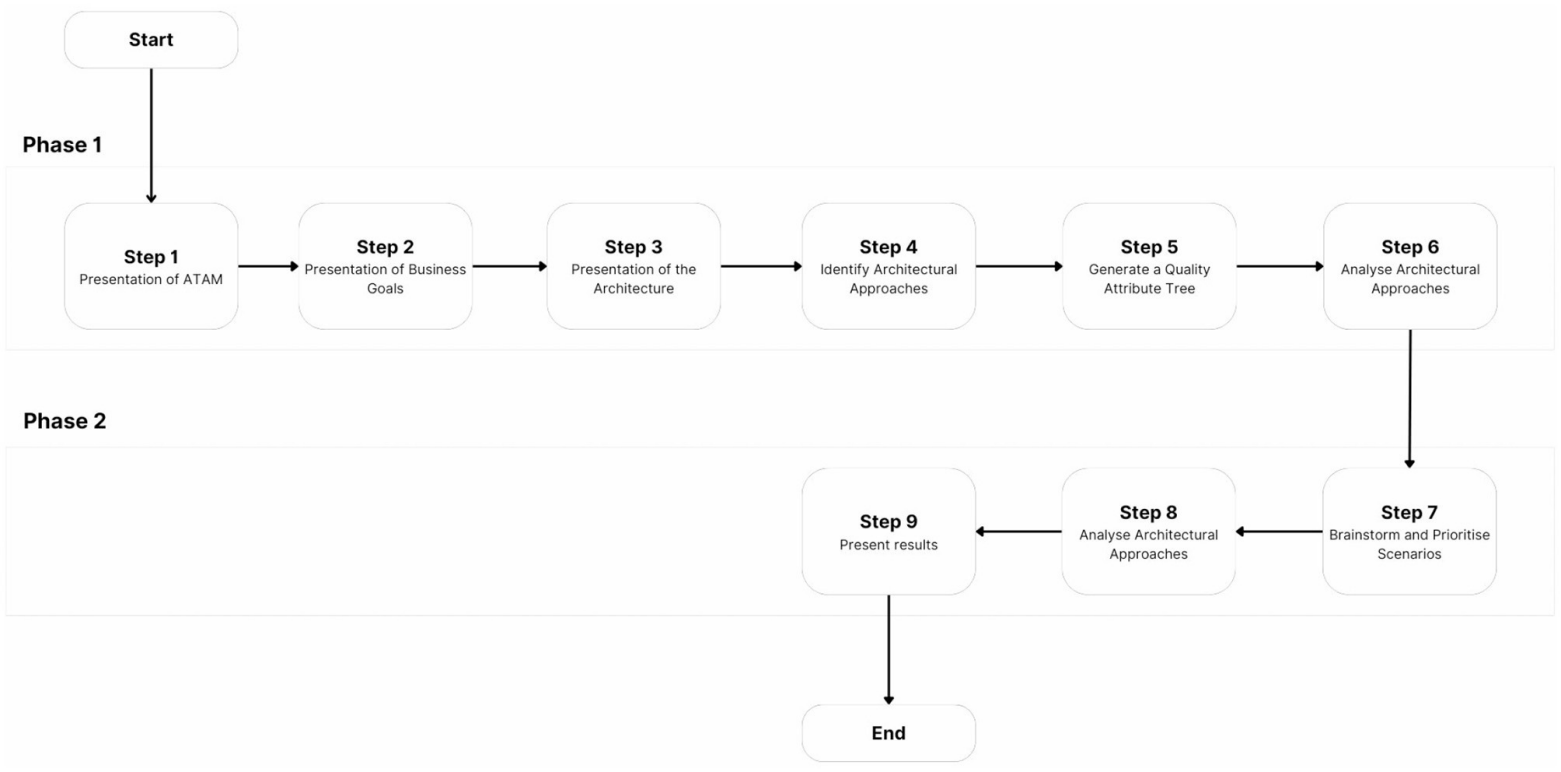
ATAM steps.

It was aimed at incorporating most components of the RA into this instance; however, logging, monitoring, service discovery, the federated governance service, and the data catalog have been omitted. Moreover, some details of this evaluation are omitted to protect the security and intellectual property of the practice, and some details are modified for academic purposes. These modifications have not affected the integrity of the evaluation. A high-level overview of the steps taken in this ATAM is portrayed in Figure 4.

### 6.1 Phase 1

This evaulation is undertaken in a subsidiary of a large-scale international company that has over 6,000 employees all around the globe. The subsidiary company offers practice management software for veterinary practitioners via Software as a Service (Saas) and has over 15,000 customers from the USA, UK, Australia, New Zealand, Canada, Singapore, and Ireland, among which are some of the biggest equine hospitals, universities, and veterinary practices. The company is currently at the stage of shifting from a centralized synchronous architecture into a decentralized event-driven microservices architecture and is ambitious to adopt artificial intelligence and BD.

The initial step was the identification of relevant stakeholders. For this purpose, the key stakeholders in the company's technical governance team are approached. The aim was to incorporate at least two lead architects of the company in this process. The emphasis was on architects who have been in business for a long period of time. This was to ensure that no important element was missed in the process of evaluation. As a result, two lead development architects were invited, along with the head of product and a business analyst, for phase 1.

#### 6.1.1 Step 1 and 2: introduction

During the initial meeting, in step 1, ATAM was presented with a clear description of its purposes. In step 2, stakeholders discussed the background of the business, some of the challenges faced, the current state of affairs, the primary business goals, and architecturally significant requirements. This step illuminated integral elements such as (1) the most important functions of the system; (2) any political, regional, or managerial constraints; (3) the business context and how it relates to our prototype; and (4) architectural drivers.

#### 6.1.2 Step 3: present the architecture

In step 3, the prototype has been presented, our assumptions have been stated, and variability points have been portrayed.

#### 6.1.3 Step 4: identifying architectural approaches

To establish the architectural styles, the prototype was first analyzed with regard to the architectural patterns and principles depicted in Section 5.2. A deeper analysis was then conducted, and architectural decisions were justified. The event-driven nature of the prototype was discussed, and the usefulness of the domains was discussed.

#### 6.1.4 Step 5: utility tree elicitation

To generate the utility tree, the most important quality attributes first had to be learnt. While these quality attributes were learned about in step 2 shortly, in this step they were probed deeper. Assumptions were first presented and double-checked with the stakeholders. Whereas concerns over privacy were raised by some stakeholders, the members unanimously agreed that performance, availability, and maintainability are the most important quality attributes. This was in line with our assumptions. In this process, the technical difficulty was rated, and the business importance was rated by the key stakeholders.

**Figure 5:**
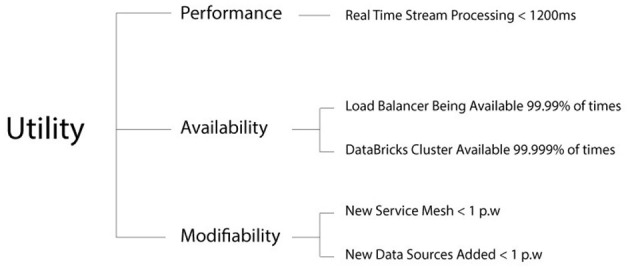
Utility tree.

Based on these premises, the utility tree has been generated (Figure 5). Each node on the utility tree corresponds to specific real-world scenarios, linking quality attributes to measurable outcomes. This ensures that performance, availability, and modifiability are tested against the day-to-day operations of the SaaS environment.

In addition to key quality attributes being identified, specific scenarios were elicited to test these attributes within the context of our SaaS company's operational environment. Each scenario was designed to rigorously test the Cybermycelium architecture against the metrics defined in the utility tree. The scenarios include the following:

- Scenario 1: real-time processing of streaming data from multiple clinics, with the system maintaining response times under 1,200 ms even during peak usage.
- Scenario 2: simulated failure of a data center to test the resilience of the load balancer and the cluster availability, maintaining 99.99 and 99.999% uptime, respectively.
- Scenario 3: integration of a new third-party service within a week, showcasing the modifiability and extensibility of the service mesh infrastructure.
- Scenario 4: addition of new data sources in response to changing privacy regulations, completed within the modifiability goal of less than one week.

These scenarios provide a broad overview of the types of challenges and requirements the architecture must address. They serve as foundational concepts that will be further refined into more specific and detailed scenarios in subsequent steps of the ATAM process.

#### 6.1.5 Step 6: analyse architectural approaches

Prior to commencing this step, simulations of the prioritized scenarios were conducted against the prototype to validate the architecture's response against the utility tree metrics. Valuable insights into the system's performance under various stress conditions and its ability to adapt to new requirements rapidly were provided by these simulations.

After this, analysis of the architectural approaches took place. In this step, the lead architects were asked to probe the architecture, and an explanation of how the prototype is addressing each scenario was provided. The architectural constructs were justified by evaluating key quality attributes previously collected for the purposes of this evaluation. The following was explained for each quality attribute:

- For performance, Nginx, Kafka, Istio, DataBricks, and the AWS Application Load Balancer have been described.
- For availability, Kafka, Event Archive, Nginx, Controllers, Data Lake, and Istio have been discussed.
- For modifiability, the concept of domain-driven design, the service mesh, zero coupling, the plug-and-play nature of the archetype, the ability to add desireable services through event brokers, and the distributed nature of the architecture have been discussed.

The result of this step was the identification of sensitivity points, trade-offs, risks, and non-risks. This step took longer than anticipated as a variety of questions arose and many aspects of the architecture were challenged. The details are discussed in Section 6.2.3.

### 6.2 Phase 2

This phase was a more serious phase of the evaluation, as more stakeholders were invited, more scenarios were collected, and even simulations were created. For this phase, in addition to lead architects, a product owner responsible for the product in which the artifact is tested, a quality assurance engineer, and several developers were invited. Step 1 was repeated, a recap of steps 2–6 was provided, and the current list of risks, non-risks, sensitivity points, and trade-offs was shared.

This phase is an iteration of phase one, so scenarios were collected, architectural approaches were analyzed, and finally, the result of the evaluation was presented.

#### 6.2.1 Step 7: brainstorm and prioritize scenarios

Building upon the high-level scenarios defined in Step 5, this step involves a detailed brainstorming session to develop specific, context-driven scenarios. These refined scenarios provide a fine-grained and realistic set of challenges and opportunities that the Cybermycelium architecture must address, reflecting the unique operational environment of the SaaS company. They are designed to be direct derivatives of the broader concepts introduced earlier, now tailored to test the system's responses in a more rigorous and precise manner.

Based on this premise, in this step, stakeholders were asked to come up with three different kinds of scenarios: growth scenarios (anticipated changes), use-case scenarios (typical usage of the system), and exploratory scenarios (extreme cases). Twenty scenarios were created as a result of this, which the stakeholders were then asked to vote on.

Drawing from the quality attributes highlighted in the utility tree, stakeholders were prompted to conceive scenarios that test the system's capabilities in a pragmatic setting. The following scenarios were derived, each tailored to challenge and assess the architecture's response to realistic operational demands:

- Scenario 1: “Rapid diagnostics turnaround”—in the context of a veterinary hospital, the architecture must support the real-time analysis of lab results, enabling a diagnosis for conditions such as Lyme disease within a critical time window and ensuring that response times remain below the threshold of 1,200 ms.
- Scenario 2: “Disaster recovery”—a simulation of a regional data center outage tests the system's failover mechanisms, specifically the ability of the load balancer and data cluster to maintain operational availability above 99.99%.
- Scenario 3: “Seamless integration”—the architecture must facilitate the integration of a new third-party service mesh within a week, demonstrating the system's adaptability to evolving business partnerships and technical ecosystems.
- Scenario 4: “Compliance adaptation”—in response to updated privacy regulations, the system must accommodate the addition of new data sources and changes to data handling processes within a week, showcasing the architecture's modifiability and compliance agility.

In turn, these scenarios are described as two user journeys:

- A cat owner brings a cat to the veterinary hospital. The cat has symptoms of Lyme disease and should be diagnosed in a timely manner to avoid master problems.
- There have been numerous cases of cancer in pets in certain environments. This environment should be analyzed to see if environmental factors play a cancer inducing role.

#### 6.2.2 Step 8: analyse architectural approaches

Before starting this step, a few days of rest were taken to simulate the scenarios against the prototype. While ATAM does not prescribe this, the evaluation was augmented with this simulation to ensure that no necessary architectural detail was overlooked. This improved confidence in the RA and the architectural probing questions to come.

The scenarios were emulated against the prototype by creating relevant topics in the Kafka, having the data flow, having the ingress in the service mesh digest it and flow it into the storage and processing, and so on and so forth. Real-world data have been used, so there was no need for data fabrication and synthesis. Nginx was configured to pass the request to the responsible Lambdas, and Lambdas then produced the necessary events and sent them to Kafka.

The simulation was presented alongside some metrics captured and displayed in the cloud served Garafana instance. From here on, this step followed the exact same procedure as step 6, with the difference that this time there had been more extensive probing and analysis of the architecture and the simulated scenarios. The simulation and results helped clarify some of the architectural constructs and led to the emergence of several questions:

- How does the system recover if the event backbone goes out of order?
- What if the service mesh ingress is not available?
- Should privacy be its own service? or should it sit in federation?
- Should we have a dedicated service mesh for metadata management?
- How easy is it to extend and modify current services?
- Should there be a certain order to events?
- Is there a benefit to creating event mesh between event brokers?
- Where is the best place to scrub sensitive data from the incoming streams?

#### 6.2.3 Step 9: present results

In this last step, the collected theories form the process of evaluation, discussed in terms of quality attributes, risks, sensitivity points, trade-offs, and other unplanned discussions that arose during the meetings.

Based on the result of our evaluation, stakeholder feedback, the utility tree, and the architectural qualities of Cybermycelium, it is deduced that system quality Q_S_ is a function f of the quality attributes availability Q_A_, performance Q_P_, and modifiability Q_M_.


(1) $$\begin{array}{l} Q_{\text{S}}=f\left(Q_{\text{M}},Q_{\text{A}},Q_{\text{P}}\right) \end{array}$$

##### 6.2.3.1 Performance

To analyse the approach in line with the utility tree, after the simulated scenarios had been created, a cloud stress testing agent (StressStimulus) was used. After this stress test had been run a couple of times, it became evident that the cold start latency of AWS Lambda services can affect the performance requirements stated in the utility tree. Anywhere from 100 ms to over a second can be taken by a Lambda at a cold start time. This latency varies and is hard to nail down, but even considering the latency, an average of 1,000 ms of response time has been captured from the system, which is in line with the utility tree. While this issue could be solved by replacing Lambdas with EC2s or Fargates, the cost would be increased, the maintainability of the architecture would be affected (a server has to be provisioned and maintained), and a rework of several services would be required.

In addition, other Lambda like solutions exist that have actually solved the cold start problem; one good example is the cloud workers offered by CloudFlare. However, a multi-cloud approach is not yet open to the company chosen for the purposes of this evaluation, and thus, AWS is the only option. Moreover, predictable start-ups with provisioned concurrency could be implemented but that requires more effort and is outside the scope of this study. As the architecture is distributed, the latency in between services has also been measured as tail latency is a known issue in distributed systems. Due to the fact that the service mesh was hosted on a private network on a virtual cloud, no major issue could be found with cloud latency, and the average response time was under 1,000 ms. A streaming process was implemented in Databricks; it was opted not to use micro-batch to have an accurate evaluation, and it was decided not to configure the fair scheduling pool so as to test the worst case scenario.

After creating and analyzing various performance models of the system, it has become clear to us that latency, side effects such as input and output, and mutations and transformations were the most important performance sensitivity points. Our performance model was built based on the following cases:

- Periodic, regular data dispatch to the product domain.

The event-driven nature of the system really helped with handling throughput and concurrency. Whereas there have been bottlenecks in the areas of storage and network latency, the system has managed to reach its desired performance on average. Given this insight and after some rigorous testing, the system's performance sensitivity is characterized as follows:


(2) $$\begin{array}{l} Q_{\text{P}}=h\left(s,l,cbp\right) \end{array}$$

That is, the system is sensitive to side effects (s), latency (l), and concurrency back pressure (cbp).

##### 6.2.3.2 Availablity

As guided by the utility tree, the key stimulus to model for the prototype is the failure of the ingress (load balancer), the data processing cluster, and most importantly, the event backbone. Due to the distributed nature of Cybermycelium and the derived prototype, failure in one service, if not handled properly, can have a ripple effect on the system. This is one area where the idea of “event brokers” was found to be really helpful. By implementing circuit breakers in event brokers, other nodes of the system were prevented from being affected by the failure of one. The events that the node was about to receive before it failed were also archived.

Whereas the event archive has played an ancillary role in providing archive to various circuit breakers, its main functionality was to provide event history to the event backbone in the event of failure. This is again achieved by circuit breaking at the broker level and event retrieval from the event archive. On the other hand, in relation to container orchestration and health checks, Kubernetes provided a declarative API to handle the state of the system. With setting replica sets and necessary deployments, the master node kept ensuring that a certain number of pods were always available. This implies that it is critical for the master node to be available at all times.

Based on these findings, the system's availability is characterized as follows (g is the fraction of time that the system is working):


(3) $$\begin{array}{l} Q_{\text{A}}=g\left(\lambda_{\text{E}},\mu_{\text{C}},\mu_{\text{S}}\right) \end{array}$$

That is, system availability is primarily affected by the failure rate of the event backbone (λ_E_), the time it takes for the circuit breaker to trip and become available again (μ_C_), and the time it takes for the service to recover from failure (μ_S_).

One major factor that really helped alleviate many issues with distributed systems was the cloud-native aspect of Cybermycelium. Whereas this aspect of the architect has not been discussed previously, the prototype was easily deployed in AWS with well-known Amazon web services. As on-premise data centers were not handled, much of the hardware was handled by the cloud company.

##### 6.2.3.3 Modifiability

To analyse modifiability, the guidelines of SAAM (Kazman et al., 1994) were followed. The distributed and service driven nature of the prototype allowed us to easily achieve the utility tree and even more. All of our cloud based infrastructure has been written as Terraform code in HCL, which means adding a new node to the system was as easy as copying the worker groups block in the EKS configuration and setting its hardware properties. Different services and deployments could then be easily deployed and have the public Docker images run. Brokers were also streamlined, and a new broker could be spun up within minutes. One area that was found a bit challenging to modify was perhaps the Databricks cluster and the EKS ALB ingress (Nginx).

Certification management was also easily handled through Istio, local CertManager, and Let's Encrypt. One area that could be taking a bit longer was the inclusion of private Docker image secrets as a Kubernetes secret and having it refreshed every 12 h. To the best of our knowledge, cron jobs were the only way to achieve this, but the implementation was not straight forward.

On the other hand, bringing up a scalable Kafka cluster was not that difficult, but there were so many configurations that one could choose to turn on or amend. This can potentially affect modifiability in the long run, when the company might have varying and sometimes conflicting requirements.

Modifiability is also affected by the skillset of the engineers and how familiar they are with Kubernetes, Databricks, and Istio. Taking all these into consideration, the system's modifiability is characterized as follows (s is the skill set required):


(4) $$\begin{array}{l} Q_{\text{M}}=s\left(K,D,K\right) \end{array}$$

That is, the system modifiability is affected by Kubernetes maintenance (K), Databricks maintenance, versioning, and configuration (D), and Kafka versioning, maintenance, and configuration.

##### 6.2.3.4 Trade-off points

As a result of these analyses, two trade-off points are identified:

1. Event backbone and event brokers.
2. Service mesh.

One area that has raised many worries is the event backbone. The event backbone being the communication facilitator has raised a lot of questions, and many are worried that this might turn into a bloated architectural component such as the enterprise service bus (ESB) in service-oriented architectures (SOAs). Many of these questions and issues were addressed both in the discussion and the prototype. By implementing an event archive, it meant that if the event backbone went down, the previous state of affairs could be restored and services could be brought to the correct state. The implementation of circuit breakers through the event brokers further solidified the availability of the architecture and could be deemed to affect reliability too. Along these lines, event brokers helped us address some of the modifiability challenges. Having these event brokers setup means that different environments do not implement their own event processing mechanisms, and the interface is unified across them. This clear interface contributed positively to the overall modifiability of the system and allowed engineers to simply copy the broker for their services. In addition, brokers also improved interoperability, and hard-to-trace bugs led to processor missmatches.

Given all, Cybermycelium does not tend to dictate what has to be done or kill the creativity of the archites but rather aims to shed light on a novel perspective on designing BD systems. Therefore, the event backbone and event brokers introduce a trade-off between performance, availability, and reliability. Whereas eliminating the event backbone may increase availability longitudinally and increase modifiability cross-sectionally, it may affect the performance quality attribute in a negative way. This is due to the fact that the event backbone is distributed in nature, can scale well to account for demans, can cache and remember communication paths, merge event streams, provide windowing techniques, and be configured to facilitate certain access patterns that are common to the system.

Another area where stakeholders were challenged was the idea of service mesh. Whereas this makes a lot of sense to developers who had to figure out how the twisted platform worked, the benefit perhaps was not that evident to everyone from the beginning. This is another area of trade-off. While having the service mesh affects the modifiability of the system in a negative way from a platform point of view, it does increase it from a data engineering and software engineer point of view. The service mesh may also affect performance slightly, but the effect is negligible. Service mesh also affects availability positively by streamlining the platform interfaces, providing an orchestrator (control tower), and doing health checks through proxies.

##### 6.2.3.5 Limitations

Cybermycelium is a new perspective on BD system development and tends to absorb many of the well-established patterns and ideas from various domains. Being distributed in nature, there are still many areas in which Cybermycelium can be improved. For instance, a great answer to tail latency issues, which can affect the system negatively, is still not available. In addition to that, feedback has been received that many developers find Cybermycelium a complex architecture that requires a lot of skill to implement. It requires an understanding of event-driven systems, event streaming, service meshing, cloud computing, and even data mesh. It is not thought that a modern distributed BD architecture should be simple, but it is strived to simplify the ways in which Cybermycelium can be absorbed.

Taking all these into consideration, it is posited that distributed BD systems are still in their infancy stage, and much work is required to facilitate this area of research. This research could be in the areas of BD distributed patterns, event-driven BD systems, data mesh, BD RAs, and methods for creating BD distributed architectures.

Moreover, the security, privacy, and metadata aspects of BD need substantial work at the macro- and micro-levels. More mature technologies and better architecture that combine these technologies into a solution are needed. This is one major area on the roadmap.

##### 6.2.3.6 Threats to validity

This section acknowledges the limitations and potential biases inherent in the evaluation process and the results thereof, aiming to provide a balanced and realistic interpretation of the findings.

In conducting the evaluation of the Cybermycelium architecture using the ATAM method, several threats to validity need to be considered:

1. Selection bias: the scenarios and stakeholders involved in the evaluation were chosen from a specific context, which may not represent all possible use cases and viewpoints. This selection bias might limit the generalizability of the findings to other contexts or architectural needs.
2. Evaluator bias: as the evaluators are also the architects of the system, there is an inherent risk of confirmation bias, where evaluators might favor findings that confirm the architecture's intended benefits. Third-party validation or blind evaluations can mitigate this risk.
3. Scenario validity: the scenarios used in the utility tree elicitation and subsequent steps may not capture the complexity or unpredictability of real-world operations. While they proxy actual system behavior, they may overlook aspects.
4. Technological evolution: the chosen technologies and tools are subject to rapid evolution and change. The evaluation's relevance may diminish as new technologies emerge or existing ones evolve, affecting the architecture's performance, availability, and modifiability.
5. Complexity and scale: the distributed nature of the Cybermycelium architecture adds layers of complexity that might not be fully addressed or understood in the evaluation. The scale at which the system operates can introduce unforeseen challenges not captured in the evaluation.

Recognizing these threats is crucial for interpreting the evaluation results in the right context. It is also important for future work to continuously validate and refine the architecture, considering these limitations and the evolving nature of technology and business needs.

## 7 Discussion

This study introduced Cybermycelium, a novel domain-driven distributed reference architecture for BD systems, designed to address limitations in current BD architectures. Motivated by the challenges of monolithic data pipelines and siloed data engineering teams, Cybermycelium proposes a decentralized approach that leverages principles from domain-driven design, event-driven architecture, and microservices. The architecture aims to improve scalability, flexibility, and data governance in BD systems.

The research employed a systematic methodology, combining a comprehensive literature review with architectural design and evaluation using the ATAM. A prototype implementation in a real-world organizational context allowed for practical assessment of Cybermycelium's performance, availability, and modifiability. The evaluation revealed promising capabilities in handling high data volumes and velocities, with the event-driven nature of the architecture contributing to system resilience and scalability.

Key findings from the evaluation include the identification of trade-offs between system flexibility and complexity, the critical role of the event backbone in system performance, and the importance of specialized technical expertise in implementing and maintaining the architecture. The study also highlighted the potential of Cybermycelium in addressing data governance challenges through its federated governance service, a feature that aligns with increasing regulatory requirements in BD management.

### 7.1 Why Cybermycelium?

If our aspiration to enhance every business aspect with data needs to come to fruition, we need a different approach to data architecture. Traditional data warehouse approaches to business intelligence, while addressing the volume and computing aspects of data, have failed to address other characteristics of it: heterogeneity and proliferation of data sources (variety), the speed at which data arrive and need to be processed (velocity), the rate at which data mutate (variability), and the truth or quality of the data (veracity).

Integral to the success of any BD initiative is the underlying data architecture that governs the entire system, its components, their relations to each other, data flow, and the principles and standards that govern the quality attributes and evolution of the system (Serra, 2024). This architecture and design process, if done underlying current prevalent approaches, can result in losses and may leave managers disappointed. Nevertheless, it is not suggested that all these architectures will fail; perhaps some have proven to be successful in a specific context. There are two threats to the maintainability and scalability of these systems: (1) Data source proliferation: as the BD system grows and more data sources are added, the ability to ingest, process, and harmonize all these data in one place diminishes, (2) Data consumer proliferation: as the variability of the data rises, the sum of aggregations, projections, and slices increases, which in turn adds more work to the backlog of the data engineering team, slowing down the process of serving the data to consumers.

Currently, BD RA architectures are usually segregated into pipelines that process data differently. While each pipeline has its own responsibility to handle various aspects of the BD system, there is still a high level of coupling between the pipelines. This coupling is even more highlighted when the company is at the stage of rapid experimentation with data sources and would like to explore new domains of insight generation, and this in turn means that delivering new features and values is orthogonal to the axis of change.

Another major issue with the current architectural approaches is that data engineering is usually confined to a team of hyper-specialized individuals who are siloed from the operational units of the organization. These teams, being fully responsible for creating the infrastructure for data processing, are often absent in business knowledge and the domain, which limits their productivity.

In the following section, a comparison of Cybermycelium and current existing RAs is explored. This is to position this study in academia and highlight the contribution and the architectural evolution that this artifact provides.

### 7.2 A comparative analysis

This section offers a comparison of Cybermycelium with existing BD RAs, emphasizing its differences in areas such as data processing, scalability, security, privacy, data management, and adaptability. The following sub-sections start by describing a common BD RA and then compare it to Cybermycelium.

#### 7.2.1 Data processing and scalability

Lambda Architecture, as presented by Kiran et al. (2015), and Kappa Architecture, discussed in Kreps (2014), are both designed to handle data processing in big data systems. Lambda Architecture uses a dual-layered processing framework to handle batch and real-time streaming data, while Kappa Architecture simplifies this by treating all data as a single stream and merging the processing layers.

However, both Lambda and Kappa architectures focus primarily on the data processing aspect and do not extensively address cross-cutting concerns such as data governance, security, or metadata management. They provide simple, high-level views of data processing but lack a comprehensive framework for handling the diverse needs of modern data-driven organizations.

In contrast, Cybermycelium takes a more holistic approach to data processing and scalability. It separates batch and stream processing using dedicated controllers, similar to Lambda Architecture, but also incorporates additional components to address cross-cutting concerns. For example, Cybermycelium includes a federated governance service for data governance and compliance, a metadata catalog for data discovery and lineage tracking, and services for data product sharing and automated infrastructure provisioning.

#### 7.2.2 Security and privacy

From a security and privacy point of view, most RAs found in the studies of Ataei and Litchfield (2022) are lacking. The Intel Healthcare RA (Sikora-Wohlfeld et al., 2014), designed for healthcare data processing, exhibits its limitations in its focus on security and privacy, which are critical in handling sensitive health data. Similarly, IBM's Healthcare RA (Quintero and Lee, 2019), while effective in its core functionality, does not prioritize security and privacy aspects sufficiently.

In contrast, Cybermycelium introduces architectural constructs that can significantly improve security and privacy from an architectural standpoint. The domain-driven design and decentralized data ownership model, inspired by Dehghani (2022), allows for more granular access control and data governance. By treating data as a product and assigning ownership to specific domains, Cybermycelium enables domain teams to implement security measures and access controls tailored to their specific data assets and compliance requirements.

Moreover, the federated governance service in Cybermycelium provides a framework for enforcing consistent security policies and privacy standards across domains. This service can be used to define and monitor compliance with data protection regulations, such as HIPAA or GDPR, ensuring that sensitive data are handled appropriately throughout the organization.

#### 7.2.3 Data management and quality

Oracle's RA (Cackett, 2013), although robust in its overall structure, falls short in emphasizing data quality and metadata management. Cybermycelium fills this gap with a metadata management system that ensures data quality and consistency across its lifecycle. This system includes mechanisms for data validation, cleansing, and enrichment, providing a higher level of assurance in data quality and usability.

Moreover, Maier's RA (Maier et al., 2013), despite its comprehensive approach to BD, does not sufficiently address dynamic data quality in diverse environments. Cybermycelium addresses this limitation by implementing robust data quality mechanisms. These mechanisms are designed to maintain high data integrity across varying datasets and use cases, ensuring that the data are accurate, complete, and relevant for analytical purposes.

#### 7.2.4 Customization and industry adaptability

Microsoft BD Ecosystem RA (Levin, 2013) offers a structured approach to BD processing within the Microsoft suite of tools. However, its adaptability to varying industry-specific needs and non-Microsoft ecosystems is limited. Cybermycelium addresses this by adopting a technology-agnostic design, ensuring compatibility and flexibility across different platforms and tools. It facilitates customization to align with the specific requirements of diverse industries, from healthcare to retail, by allowing integration with various data sources and processing tools, thus enabling more tailored and effective BD solutions.

#### 7.2.5 Innovation in data storage and handling

The Data Lake Approach (Pääkkönen and Pakkala, 2015), while providing a consolidated platform for data storage, often leads to challenges in data governance, potentially transforming data lakes into unmanageable “data swamps.” Cybermycelium confronts this issue with a decentralized data governance model, which empowers individual domains to manage and govern their data while still adhering to overarching organizational policies. This approach enhances data quality, accessibility, and compliance.

Moreover, the NIST BD RA (Chang and Boyd, 2018), though comprehensive in scope, often lacks specificity in its practical application, particularly in data storage and handling strategies. Cybermycelium introduces an innovative approach with its distributed data mesh. This mesh not only facilitates efficient data management but also supports scalability and interoperability across various business domains, thereby offering a more practical and application-focussed data architecture.

#### 7.2.6 Cloud-based and decentralized architectures

Cloud-based solutions, represented by architectures such as Amazon Web Services or Microsoft Azure, offer scalability and flexibility but tend to maintain a centralized, monolithic structure in data processing and storage. This structure can limit the system's responsiveness to changing data demands and hinder rapid scalability.

Cybermycelium, while leveraging the benefits of cloud infrastructure, such as on-demand resource allocation and global accessibility, diverges from the traditional cloud-based approach by embracing a decentralized, domain-driven architecture. This design choice promotes modularity, making each component of the architecture independently scalable and adaptable. It enables Cybermycelium to avoid the limitations typically associated with monolithic cloud-based systems, thus offering a more dynamic and responsive BD architecture. An overview of the comparative analysis of Cybermycelium with existing BD RAs is presented in Table 3.

**Table 3:** Comparative analysis of Cybermycelium with existing big data reference architectures.

**Area of comparison**	**Limitations of existing BD RAs**	**Cybermycelium's approach**
Data processing and scalability	Focus on data processing	Holistic approach
	Lack comprehensive framework	Addresses cross-cutting concerns
	Tightly coupled layers	Microservices-based architecture
Security and privacy	Limited focus on security and privacy	Domain-driven design
	Insufficient data protection	Federated governance, modular design
Data management and quality	Insufficient emphasis on data quality	Metadata management system
	Limited ability to handle dynamic quality	Robust data quality mechanisms
Customization and industry adaptability	Limited adaptability to industry-specific	Technology-agnostic design
	needs and non-proprietary ecosystems	Facilitates customization
Data storage and handling	Challenges in data governance	Decentralized governance model
	Lack of specificity in application	Distributed data mesh
Cloud-based and Decentralized architectures	Centralized, monolithic structure	Decentralized, domain-driven
	Limits responsiveness and scalability	Modular components for adaptability

### 7.3 Broader implications and adoption considerations

The implementation of Cybermycelium represents a paradigm shift in big data architecture, aligning with emerging concepts such as data mesh (Dehghani, 2022) and data fabric (Gartner, 2021). This alignment positions Cybermycelium at the forefront of decentralized data management strategies, emphasizing domain-oriented, distributed architectural patterns.

Adopting Cybermycelium necessitates significant organizational restructuring, transitioning from traditional siloed approaches to cross-functional, domain-driven teams. This transformation echoes the sociotechnical systems theory (Baxter and Sommerville, 2011), which emphasizes the interdependence of social and technical factors in organizational change. New roles such as data product owners become essential, reflecting a shift toward treating data as a product (Levy and Wilensky, 2021). The technical implementation of Cybermycelium requires a diverse skill set, encompassing proficiencies in event-driven architectures, distributed systems, and domain-driven design. This multidisciplinary approach aligns with the T-shaped skill concept (Guest, 1991), where professionals possess both depth in specific areas and breadth across related domains.

While Cybermycelium offers potential benefits in scalability, flexibility, and data governance, its adoption faces several challenges. These include the technical complexity inherent in distributed systems, organizational resistance to change, and initial financial investments. These challenges are consistent with the innovation diffusion theory (Rogers, 2003), which posits that the adoption of new technologies is influenced by their perceived attributes and the social system in which they are introduced.

To mitigate these challenges, organizations can employ strategies such as phased implementation and comprehensive training programmes. These approaches are rooted in change management theories such as Kotter's 8-step model (Kotter, 1996), emphasizing the importance of creating a sense of urgency, building a guiding coalition, and anchoring new approaches in the organizational culture.

### 7.4 Limitations and future work

Cybermycelium, being a new perspective on BD system development, still has areas for improvement. The distributed nature of the architecture presents challenges, particularly in addressing tail latency issues. The complexity of the architecture requires a high level of skill to implement, necessitating understanding of event-driven systems, event streaming, service meshing, cloud computing, and data mesh concepts.

Future research directions could include the following:

- Investigating practical implementation and integration of Cybermycelium in diverse organizational contexts.
- Assessing its efficacy in handling emerging data formats and processing paradigms.
- Refining the architecture based on real-world performance metrics and evolving industry trends.
- Developing guidelines and best practices for adopting and maintaining decentralized, domain-driven BD architectures.
- Exploring integration patterns to help companies transition from centralized to emergent distributed BD architectures.
- Investigating the use of Cybermycelium for new AI applications using Large Language Models (LLMs).

## 8 Conclusion

This study introduces Cybermycelium, a novel domain-driven distributed reference architecture for Big Data systems, addressing critical limitations in current architectures. Cybermycelium's key contributions lie in its decentralized, event-driven approach, integrating principles from domain-driven design and microservices. It enhances scalability, flexibility, and data governance while introducing a federated governance model that balances domain autonomy with organizational oversight.

The practical evaluation using ATAM provides valuable insights into Cybermycelium's performance, availability, and modifiability in real-world scenarios. This evaluation also highlights important trade-offs and challenges in implementing distributed Big Data architectures, contributing to the broader discourse on Big Data system design. Cybermycelium's alignment with emerging concepts such as data mesh and data fabric positions it at the forefront of Big Data management strategies. By offering a more adaptable solution to the increasing volume, variety, and velocity of data, it addresses the limitations of monolithic data pipelines and siloed data engineering teams.

While implementing Cybermycelium presents challenges, including organizational restructuring and the need for diverse technical skills, its potential benefits in improved data quality and more agile decision-making are substantial. As Big Data continues to evolve alongside AI and machine learning advancements, architectures such as Cybermycelium will be crucial in enabling organizations to effectively harness their data assets. In conclusion, Cybermycelium offers a promising direction for Big Data architecture, providing a flexible, scalable, and governance-focussed framework adaptable to the changing needs of data-driven organizations. Its contributions open new avenues for research and practical implementation, furthering the field's ongoing development and the realization of Big Data's full potential.

## Data Availability

The original contributions presented in the study are included in the article/supplementary material, further inquiries can be directed to the corresponding author.
